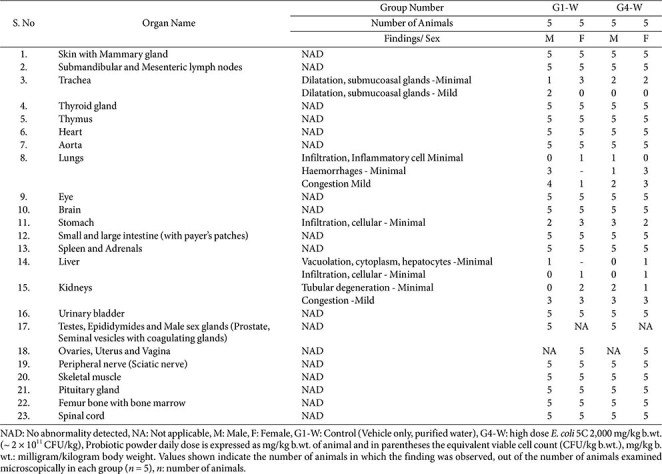# Subacute 28-Day OECD-Guided Oral Toxicity Study of *Escherichia coli* 5C (LMG S-33222) in Wistar Rats and Immunocompromised Nude Mice

**DOI:** 10.4014/jmb.2512.12003

**Published:** 2026-01-26

**Authors:** Francesco Di Pierro, Amjad Khan, Gayathri Veeraraghavan, Kalaivani Periyathambi, Sathiya Ravikumar, Maria Laura Tanda, Nicola Zerbinati, Andrea Carugno, Ikram Ujjan

**Affiliations:** 1 Microbiota International Clinical Society, Torino, Italy; 2 Scientific & Research Department, Velleja Research, Milano, Italy; 3 Department of Medicine and Technological Innovation, University of Insubria, Varese, Italy; 4 Department of Biochemistry, Liaquat University of Medical & Health Sciences (LUMHS), Jamshoro, Pakistan; 5 Nuffield Division of Clinical Laboratory Sciences (NDCLS), University of Oxford, UK; 6 Centre For Toxicology and Developmental Research (CEFTE), Sri Ramachandra Institute of Higher Education and Research (DU), Porur, Chennai, India; 7 Endocrine Unit, Department of Medicine and Surgery, University of Insubria, Varese, Italy; 8 Dermatology Unit, Department of Medicine and Surgery, University of Insubria, Varese, Italy; 9 Department of Pathology, Liaquat University of Medical & Health Sciences (LUMHS), Jamshoro, Pakistan

**Keywords:** *Escherichia coli* 5C, LMG S-33222, Probiotic safety, Repeated-dose oral toxicity, OECD 407, Wistar rats, Nude mice, Immunocompromised model, NOAEL, Non-pathogenic *E. coli*

## Abstract

Ensuring the safety of microbial strains intended for probiotic use is essential, particularly for species such as *Escherichia coli* (*E. coli*), which include both commensal and pathogenic lineages. *E. coli* 5C is a recently identified polyketide synthase (*pks*)-negative strain isolated from healthy infant feces and characterized as free of virulence factors, plasmids, and antimicrobial resistance, suggesting suitability as a probiotic. To confirm its *in vivo* safety, we evaluated the subacute oral toxicity of *E. coli* 5C in immunocompetent and immunocompromised rodent models. A GLP-compliant 28-day repeated-dose oral toxicity study was conducted in Wistar rats following OECD Test Guideline 407 (2008 edition), complemented by a parallel non-GLP study in athymic nude mice to assess safety under impaired immune function. Animals received purified water (control) or *E. coli* 5C at 500, 1000, or 2000 mg/kg/day (~0.5, 1, or 2 × 10^11^ CFU/kg/day), as low, mid, and high dose, respectively, daily for 28 days. Clinical signs, behaviour, body weight, feed intake, functional observations, haematology, biochemistry, urinalysis, organ weights, gross necropsy, and histopathology were evaluated. Across all treated groups in both species, *E. coli* 5C produced no mortality, morbidity, or adverse clinical effects. Haematological, biochemical, and behavioural parameters remained comparable to controls, and organ weights, gross pathology, and microscopic examinations revealed no test item-related abnormalities. No systemic infection was observed. No treatment-related adverse effects were observed at any dose level, and the highest tested dose of 2,000 mg/kg/day (2 × 10^11^ CFU/kg/day) was therefore identified as the No Observed Adverse Effect Level (NOAEL). These findings demonstrate the absence of subacute toxicity of *E. coli* 5C in both normal and immunodeficient hosts and provide a favourable preclinical safety foundation for its continued development as a candidate probiotic strain.

## Introduction

The intestinal microbiota is now recognized as a key regulator of human physiology, contributing not only to digestion and nutrient absorption but also to immune modulation, metabolic homeostasis, and even neural and oncological processes [1-5]. Maintaining a balanced microbial ecosystem (eubiosis) is therefore essential for health, and disturbances in this equilibrium have been associated with gastrointestinal and systemic disorders. Probiotic supplementation has emerged as one of the most effective strategies for restoring and maintaining eubiosis [6]. Probiotics are defined as “live microorganisms which, when administered in adequate amounts, confer a health benefit on the host” [7]. They are commonly employed to promote intestinal health, enhance mucosal immunity, and restore microbial equilibrium following dysbiosis.

Strains mainly belonging to the genera *Bifidobacterium* and *Lactobacillus* have repeatedly been shown to exert favorable effects on human health [8-10], and are widely regarded as safe probiotics[11-16]. However, probiotic safety cannot be assumed universally, particularly in vulnerable populations such as immunocompromised, pediatric, or post-surgical patients [12, 17-23]. While adverse events remain rare, they may include hypersensitivity reactions (skin rashes, itching, and swelling) [24, 25], drug–probiotic interactions [26, 27], histamine-mediated intestinal inflammatory responses [28], dissemination of antibiotic-resistance elements [29, 30], or the synthesis of potentially harmful metabolites such as colibactin — a genotoxin associated with DNA damage and colorectal carcinogenesis [31, 32]. These observations underscore the importance of comprehensive preclinical safety assessments, particularly for novel bacterial probiotics or when use is anticipated in immunocompromised hosts.

When Theodor Escherich first isolated *Escherichia coli* (*E. coli*) in 1885, it was recognized as a harmless intestinal commensal [33]. Today, this species is present in approximately 90% of healthy adults, typically representing about 1% of the gut microbial community [34, 35]. Despite this ubiquity, scientific research has historically focused on pathogenic variants, leaving commensal and probiotic lineages comparatively underexplored. Several *E. coli*-based probiotics—such as *E. coli* Nissle 1917, *E. coli* DSM 17252, and *E. coli* A0 34/86—have demonstrated clinical efficacy in gastrointestinal disorders [36-38]. However, some of these strains harbor genes associated with virulence or toxin production, such as α-hemolysin (*hly*), cytotoxic necrotizing factor-1 (*cnf1*), or the polyketide synthase (*pks*) genomic island, which encodes the biosynthetic machinery for colibactin—a genotoxin implicated in DNA damage and colorectal carcinogenesis [31, 32, 39, 40]. This underscores the importance of identifying and validating *E. coli* strains that are genetically stable, *pks*-negative, and devoid of virulence or resistance determinants.

*E. coli* 5C (deposited as LMG S-33222) is a recently isolated, non-pathogenic, *pks*-negative strain obtained from the feces of a healthy Italian infant donor [41]. Comprehensive genomic and phenotypic analyses have confirmed that this strain is plasmid-free and devoid of known virulence factors, including fimbrial operons associated with uropathogenicity, as well as antimicrobial resistance determinants [41, 42]. *In vitro* assays have demonstrated its survival under simulated gastric and intestinal conditions, strong adhesion to intestinal epithelial cells, acetate production, and balanced immunomodulatory effects involving interleukin-10 (IL-10) and interleukin-12 (IL-12) [41]. Preliminary clinical observations further indicated that oral administration of *E. coli* 5C alleviated gastrointestinal discomfort and accelerated microbiota recovery following bowel cleansing and colonoscopy [43]. The strain belongs to phylogroup A, typically associated with commensal lineages, and its probiotic properties may have clinical relevance in the management of inflammatory bowel disease, constipation, prevention of enteric as well as urinary infections, and potentially aiding in the prevention of colibactin-associated colorectal neoplasia [44-49].

**Fig. 1:**
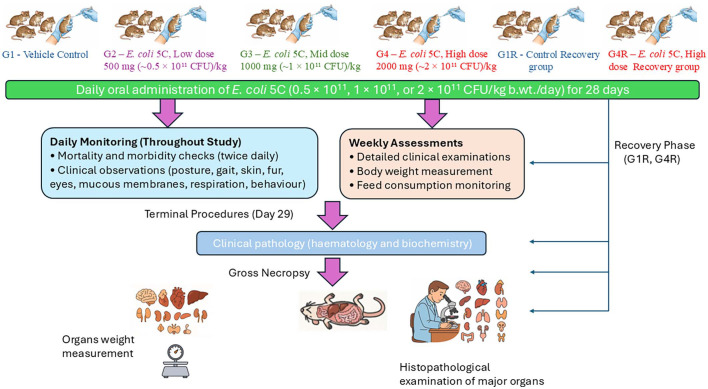
Study design and experimental workflow for the 28-day repeated-dose oral toxicity study of *Escherichia coli* 5C in Wistar rats and Athymic Nude mice, including dosing regimen, monitoring schedule, and endpoint assessments.

Given its probiotic potential and *E. coli* taxonomy, the need to ensure an adequate safety profile prompted a thorough toxicological assessment in both healthy and immunocompromised models. The present study was therefore designed to assess the subacute oral toxicity of *E. coli* 5C in accordance with the Organisation for Economic Co-operation and Development (OECD) Test Guideline (TG) No. 407 (2008 edition), *Repeated Dose 28-day Oral Toxicity Study in Rodents* [50]. Two complementary animal models were employed: (i) healthy Wistar rats under Good Laboratory Practice (GLP) conditions, and (ii) immunocompromised athymic nude mice, which provide a sensitive system for evaluating microbial behavior in hosts with impaired immune function (Fig. 1). This combined approach ensures a comprehensive safety assessment, supporting the probiotic’s further development and potential application in both healthy and at-risk populations [51].

## Materials and Methods

### Chemicals, and Reagents

All chemicals, reagents, and solvents used in this study were of analytical grade. Biochemical assay kits were obtained from Biosystems Ltd., India, electrolyte reagents and electrode packs from Roche Diagnostics, India, and haematology reagents from Mindray Medical, India. Standard rodent pelleted feed was procured from Krishna Valley Agrotech LLP Nutritional Solutions, Pune, India.

### Study Design

A tiered toxicological evaluation was conducted to assess the safety profile of *E. coli* 5C. A subacute 28-day repeated-dose oral toxicity study was performed in Wistar rats in accordance with OECD TG 407 (Repeated Dose 28-Day Oral Toxicity Study in Rodents; adopted 3 October 2008) and in compliance with the OECD Principles of GLP, as outlined in the OECD Council Decision on Mutual Acceptance of Data (ENV/MC/CHEM(98)17), to evaluate potential systemic, biochemical, and organ-specific effects following daily oral administration (Experiment 1).

A parallel, non-GLP, 28-day oral toxicity study was conducted in athymic nude mice under equivalent dosing and observational conditions (Experiment 2). Slight modifications to the test guidelines were made for the nude mouse experiment due to the immunocompromised nature of the model and the limited blood volume obtainable from individual animals; therefore, smaller analytical subsets (five animals per analysis) were used to ensure sufficient sample volume for hematological and biochemical evaluations while adhering strictly to humane limits and institutional animal welfare guidelines.

### Test Item - *Escherichia coli* 5C (LMG S-33222) Strain

The *E. coli* 5C strain used in this study was provided as a lyophilized formulation with a pale-yellow, crystalline appearance and a viable cell count of approximately 1.0 × 10^11^ CFU/g. The strain, deposited in the LMG collection of the Belgian Coordinated Collections of Microorganisms (BCCM/LMG, Belgium) under accession number, was manufactured by Teracell S.r.l. (Italy). The test item was soluble in purified water and had a measured pH of 5.43. This strain has been registered with the Italian Ministry of Health as a probiotic dietary supplement (Colipral^®^, Pharmextracta S.p.A, Pontenure, Italy, Notification No. 190304, date 17.12.2024) [52].

### Ethical Approvals

All experimental procedures were conducted at the Centre for Toxicology and Developmental Research (CEFTE), a GLP-certified facility of the Sri Ramachandra Institute of Higher Education and Research (SRIHER), Chennai, India, which is registered with the Committee for the Control and Supervision of Experiments on Animals (CCSEA; Registration No. 89/PO/ReRcBi/S/2000/CCSEA). Both the Wistar rat and athymic nude mouse studies received prior approval from the Institutional Animal Ethics Committee (IAEC), under approval numbers, IAEC/76/SRIHER/957/ 2025 and IAEC/76/SRIHER/960/2025, respectively, as well as clearance from the Institutional Biosafety Committee (IBSC Approval Nos. IBSC 2025002 for rats and IBSC 2025003 for nude mice).

### Experimental Animals and Husbandry

A total of 60 Wistar rats (both males and non-pregnant nulliparous females) and 120 healthy athymic nude mice (both sexes) were obtained from Hylasco Biotechnology Pvt. Ltd., India (CCSEA Registration No.: 1808/PO/RcBt/S/2015/CCSEA). Animals were acclimatized for five days prior to dosing, during which environmental conditions were carefully controlled. Temperature was maintained between 19.93–22.52°C, relative humidity between 42.7–65.1%, and the ventilation system provided 12–15 air changes per hour. A 12-h light / 12-h dark photoperiod was maintained using an artificial lighting cycle. Animals were housed in groups of two to three per polypropylene cage fitted with stainless steel grid tops. Autoclaved paddy husk was provided as bedding material to allow natural nesting behavior. No additional enrichment devices were used, as group housing provided sufficient social enrichment in accordance with institutional animal care guidelines. Cages were changed on alternate days, and cage grills were replaced weekly. Throughout the study, animals had ad libitum access to pelleted rodent feed and UV-irradiated purified drinking water supplied in autoclaved bottles.

### Grouping and Dose Administration

In both studies, animals were randomized into main and recovery groups using stratified body weight to ensure comparable baseline characteristics across groups. Three dose levels of *E. coli* 5C were selected based on prior short-term safety data: low dose 500 mg/kg b.wt. (~0.5 × 10^11^ CFU/kg), mid dose 1,000 mg/kg b.wt. (~1 × 10^11^ CFU/kg), and high dose 2,000 mg/kg b.wt. (~2 × 10^11^ CFU/kg), administered once daily for 28 days. Test item formulation analyses, including verification of concentration, homogeneity, and stability, were performed in accordance with GLP requirements (Data not shown).

For Experiment 1, animals were assigned to four main groups (G1-W to G4-W) and two recovery groups (G1R-W and G4R-W) (suffix -W for Wistar rats), with each group comprising 5 males and 5 non-pregnant, nulliparous females. For Experiment 2, animals were similarly assigned to four main groups (G1-N to G4-N) and two recovery groups (G1R-N and G4R-N) (suffix -N for athymic nude mice), with each group comprising 10 males and 10 non-pregnant, nulliparous females. The increased group size in the athymic nude mouse study was implemented to ensure adequate sample volumes for comprehensive hematological and clinical biochemistry analyses, given the limited blood volume obtainable per animal.

In both experiments, the control groups (G1 and G1R) received the vehicle only (purified water, 10 ml/kg b.wt.) once daily for 28 days. The treatment groups received *E. coli* 5C powder prepared in water at 500 mg/kg b.wt. (~0.5 × 10^11^ CFU/kg; low dose) in G2, 1,000 mg/kg b.wt. (~1 × 10^11^ CFU/kg; mid dose) in G3, and 2,000 mg/kg b.wt. (~2 × 10^11^ CFU/kg; high dose) in G4. High-dose recovery groups (G4R-W and G4R-N) received 2,000 mg/kg b.wt. (~2 × 10^11^ CFU/kg) once daily for 28 days, followed by a 14-day recovery period without dosing.

The grouping and dose allocations for both experiments are summarized in Table 1A (Wistar rats) and Table 1B (nude mice).

### Observations

All animals were monitored twice daily throughout acclimatization and the experimental period for mortality and morbidity. Daily clinical observations included assessment of the skin, fur, eyes, mucous membranes, respiratory patterns, posture, gait, autonomic signs (*e.g.*, lacrimation, piloerection, salivation), and the presence of any abnormal secretions or excretions. Detailed clinical examinations—encompassing home-cage, hand-held, and open-field observations—were performed prior to the first dosing and weekly thereafter until necropsy.

Individual body weights were recorded on Day 1 (prior to dosing) and weekly thereafter, including on the day of scheduled necropsy. Feed consumption was recorded weekly for all groups from Week 1 to the end of the observation period.

A Functional Observation Battery (FOB) was conducted to evaluate sensory and neuromuscular responses. Sensory reactivity to visual, auditory, and proprioceptive stimuli (*e.g.*, approach response, eye-blink reflex, touch response, forelimb and hindlimb extension, tail-pinch response, auditory startle, catalepsy, and air-righting reflex) was assessed in both species.

For Experiment 1 (Wistar rats), grip strength was measured using a calibrated grip-strength meter (three trials per animal), and locomotor activity was assessed using an automated infrared actimeter that recorded activity counts over a 3-min interval.

For Experiment 2 (athymic nude mice), an Open Field Test (OFT) was used to evaluate general locomotor activity, exploratory behaviour, and anxiety-related responses. Parameters included number of squares crossed, urination count, rearing frequency, grooming, defecation, entries into and time spent in the center zone, and time spent in peripheral zones over a 3-min interval.

### Urinalysis (Wistar Rats Only)

Urine samples were collected from Wistar rats during the final week of the study using metabolic cages with access to feed and water for ~3 to 4 h. Each sample was examined visually for physical characteristics, including colour, appearance, and volume. Semi-quantitative analyses were performed using an automated urine analyzer to assess pH, specific gravity, and biochemical constituents, including microalbumin, bilirubin, urobilinogen, ketone bodies (acetone), glucose, protein, and occult blood. In addition, microscopic examination of the urine sediment was conducted to detect the presence of epithelial cells, casts, crystals, and red or white blood cells.

### Haematology and Clinical Biochemistry

Blood samples were collected from all animals on Day 29 (main groups) and Day 43 (recovery groups) via retro-orbital sinus puncture under isoflurane anaesthesia following an overnight fast. Due to extensive analysis and limited blood volume in athymic nude mice, totally 10 animals were considered (five animals/ analysis/ group) and allocated specifically for haematological and biochemical analyses, in accordance with institutional humane limits.

Haematological parameters were measured using a Mindray haematology analyser, BC5000VET, Shenzhen Mindray Medical India Co. Ltd., India. The parameters included differential leukocyte counts, red blood cell count, haemoglobin concentration, haematocrit, mean corpuscular volume (MCV), mean corpuscular haemoglobin (MCH), mean corpuscular haemoglobin concentration (MCHC), platelet count, clotting time, and reticulocyte count.

Serum biochemical parameters were analyzed using a fully automated Biosystems biochemical analyser, A15, S.A, Spain). Evaluated parameters included glucose, total cholesterol, triglycerides, blood urea nitrogen (BUN), total protein, albumin, globulin, creatinine, urea, aspartate aminotransferase (AST), alanine aminotransferase (ALT), alkaline phosphatase (ALP), and total bilirubin. Electrolytes (sodium, potassium, and calcium) were measured using a Cobas electrolyte analyser, Roche Diagnostics, India.

### Gross Pathology and Organ Weights

At the end of the study, all surviving animals underwent necropsy following sedation with xylazine and terminal euthanasia by CO_2_ inhalation. A comprehensive gross pathological examination was conducted, including external inspection and evaluation of body orifices, followed by detailed examination of the cranial, thoracic, abdominal, and pelvic cavities and all major organs.

Absolute wet weights of the brain, heart, lungs, liver, kidneys (paired), adrenals (paired), testes (paired), epididymides (paired), prostate and seminal vesicles (with coagulating glands), ovaries (paired), and uterus were recorded. Organs and tissues were excised, weighed when appropriate, and preserved in suitable fixatives (10% neutral buffered formalin, Davidson’s, or modified Davidson’s).

### Histopathology

Histopathological evaluation was performed for animals in the control and high-dose groups (main and recovery) for both species. Tissues examined included the brain, spinal cord, pituitary gland, thyroid gland, thymus, heart, lungs, liver, kidneys, adrenal glands, spleen, stomach, small and large intestines, pancreas, reproductive organs, urinary bladder, lymph nodes, eyes, bone, skeletal muscle, and peripheral nerves; additionally, the gallbladder was examined in nude mice. Tissues were processed, embedded, sectioned, and stained with haematoxylin and eosin (H&E) for microscopic evaluation.

### Statistical Analysis

Data for body weight, feed consumption, grip strength, locomotor activity, haematology, clinical biochemistry, urinalysis, and absolute and relative organ weights were expressed as mean ± standard deviation (SD). Statistical analyses were performed using SigmaPlot (Version 14.5). Prior to inferential analysis, data were assessed for normality and homogeneity of variance. For main group comparisons (G1 vs. G2–G4), one-way ANOVA followed by Dunnett’s post hoc test was applied. For recovery groups (G1R vs. G4R), comparisons were made using unpaired t-tests. A value of *p* < 0.05 was considered statistically significant.

## Results

### Clinical Signs, Health Examination, and Mortality

**Wistar rats.** No mortality or morbidity occurred in any group throughout the study. Animals appeared healthy during daily cage-side checks and weekly detailed clinical examinations (home-cage, hand-held, and open-field assessments). No test-item–related clinical signs were observed in either sex. The skin, fur, eyes, mucous membranes, posture, gait, secretions/excretions, and handling responses were normal in all treated groups compared with their respective controls (Data not shown).

**Athymic nude mice.** No mortality or morbidity occurred in any of the control or treated groups throughout the experimental period. Daily cage-side observations and weekly detailed clinical examinations (home-cage, hand-held, and open-field) did not reveal any test-item–related abnormalities in either sex. All animals exhibited normal appearance and behaviour, with no changes in skin, fur, eyes, mucous membranes, secretions or excretions, posture, gait, or response to handling, indicating the absence of treatment-related toxicity.

### Body Weight and Feed Consumption

**Wistar rats.** There were no statistically significant differences in mean body weight between any test-item–treated groups (G2, G3, G4, and G4R) and their respective controls (G1 and G1R) in either sex (Table 2). A statistically significant decrease in feed consumption was noted in G2 males during Week 4, relative to G1. However, this change was small, isolated, and lacked dose-dependency, and was therefore considered incidental rather than treatment-related. In females, feed consumption remained comparable to controls across all groups and all weeks. Variations observed were minor, and not dose-related (Data not shown).

**Athymic nude mice.** Across all treatment groups (G2, G3, G4, G4R), there were no statistically significant differences in mean body weight in either sex when compared with their respective controls (G1 and G1R), except for a statistically significant decrease (*p* < 0.05) observed in G2 males on Day 8. This decrease was minimal, transient, and fully recovered by Day 22; therefore, it was considered incidental and not related to the test item (Table S1).

Feed consumption was generally comparable between control and treated groups across all weeks of the main and recovery periods. In males, a statistically significant decrease (*p* < 0.05) was observed in G2 during Week 4, and in G4R during Week 1, compared to their respective controls. In females, no statistically significant differences occurred in any treated group across the study duration. All variations were small, inconsistent, lacked dose-dependency, and were therefore considered not attributable to test item administration (Data not shown).

### Functional Observation Battery (FOB) and Motor Activity

**Wistar rats.** Sensory reactivity to visual, auditory, and proprioceptive stimuli remained normal across all treatment groups. All animals exhibited normal eye-blink responses, appropriate withdrawal or turning during proprioceptive testing, and mild, expected ear-flick responses to auditory stimuli.

Grip-strength and locomotor-activity measurements did not differ significantly between treated and control groups in either sex (Table 3). This indicates that *E. coli* 5C administration did not induce abnormal neurobehavioral or neuromuscular effects.

**Athymic nude mice.** Both male and female animals in the treated groups (G2, G3, G4, and G4R) exhibited normal sensory reactivity responses across all evaluated stimuli, including the approach response, eye-blink reflex, touch response, forelimb and hindlimb extension, tail-pinch response, auditory startle (click response), catalepsy, and air-righting reflex, and these responses were comparable to those of the concurrent control groups (G1 and G1R). Occasional variations, such as a mild increase in the tail-pinch response in G2 females and G3 males or a “double-tick” catalepsy score in a few animals across different groups, were also observed in control animals and were therefore considered spontaneous and not related to test-item administration.

Motor-activity parameters assessed in the open-field test—including the number of crossings, rearing, grooming episodes, time spent in the periphery, time spent in the center, and entries into the center—were likewise comparable across all treated groups (G2, G3, G4, and G4R) relative to the respective control groups (G1 and G1R). No dose-dependent or treatment-related alterations were observed (Table S2).

Overall, the FOB and motor-activity assessments demonstrated that *E. coli* 5C did not induce any neurobehavioral or neuromuscular alterations in nude mice.

### Haematology

**Wistar rats.** No test-item–related changes were observed in most haematological parameters, although a few statistically significant differences were noted in both sexes. In males, a statistically significant increase was observed in total leukocyte count in G3 and in platelet count in G3 and G4, while a statistically significant decrease in haematocrit, haemoglobin, and erythrocyte count was noted in G2, G3, and G4, compared with the G1 control group. In females, a statistically significant increase in total leukocyte count and platelet count was recorded in G3 and G4, whereas clotting time, haemoglobin, and erythrocytes were significantly decreased in G2, G3, and G4. In addition, decreases in haematocrit and eosinophils were observed in G3 and G4 compared with the G1 control group (Table 4). In the recovery groups, no statistically significant differences were observed in G4R animals of either sex when compared with the G1R control, except for a decrease in platelet count in G4R females. Although some significant increases (*e.g.*, platelet count, MCHC, total leukocytes) and decreases (*e.g.*, erythrocytes, haemoglobin, haematocrit, eosinophils, clotting time) were recorded in both sexes, all values remained within reference ranges and the laboratory’s historical control limits (Table 4a).

Overall, the findings suggest that the test item may have produced mild, dose-dependent effects on a few haematological parameters in the main groups; however, the lack of consistent dose dependency across other parameters, the absence of similar findings in both sexes, and the complete resolution of changes in the recovery groups indicate that these variations were reversible and not toxicologically significant and adverse effect.

**Athymic nude mice.** Haematological parameters in the treated groups (G2, G3, and G4) of both sexes were generally comparable with those of the control group (G1), and no treatment-related changes were identified (Table S3). A few statistically significant differences were noted, but these alterations were small in magnitude, inconsistent, and lacked dose dependency. In males, statistically significant increases in clotting time were observed in G2 and G4 when compared with G1. In females, a statistically significant increase in total erythrocyte count was noted in G2 compared with G1. In the recovery phase, decreases in haemoglobin, haematocrit, erythrocytes, and clotting time were recorded in G4R males when compared with G1R, while basophils and platelet counts were significantly lower in G4R females. Other haematological indices, including WBC and RBC-related parameters, remained within normal physiological ranges and did not show any treatment-related alterations. Despite the isolated statistical differences, all findings were within historical control limits, not dose-dependent, and lacked correlating changes in clinical pathology or histopathology, indicating that these variations were unrelated to adverse effect of administration of the test item.

### Biochemistry and Electrolytes

**Wistar rats.** Biochemistry and electrolyte values for both the main and recovery groups are presented in Table 5. In males, statistically significant decreases were observed in urea and blood urea nitrogen (BUN) across G2, G3, and G4, along with reductions in cholesterol (G4), potassium, total bilirubin (G4 and G4R), creatinine (G2), ALT (G3), and glucose (G3 and G4) when compared with the respective G1 and G1R control groups. In females, statistically significant decreases were observed in triglycerides (G2, G3, and G4) and in potassium (G4R), while significant increases in ALT and AST were noted in the G4R females when compared with the G1 and G1R controls. Despite these statistical differences, most values remained within normal physiological and laboratory historical control ranges (Table 5a). Notably, the dose-dependent reductions in urea and BUN (G2 to G4) did not correlate with changes in creatinine, and kidney histopathology did not reveal any test-item–related lesions, suggesting that these variations were transient and not attributable to the test item. Changes observed in other parameters lacked consistency between groups, were not dose-dependent, and were absent in the recovery groups. These fluctuations are therefore interpreted as biological variability rather than adverse or treatment-related effects. Overall, *E. coli* 5C did not induce any toxicologically meaningful alterations in clinical biochemistry or electrolyte profiles in either sex.

**Athymic nude mice.** Serum biochemistry values, including glucose, cholesterol, triglycerides, total bilirubin, phosphorus, urea, BUN, creatinine, liver enzymes (ALT, AST, ALP), and electrolytes (sodium, potassium, and calcium), were generally comparable between the control groups (G1 and G1R) and the treated groups (G2, G3, G4, and G4R) across both sexes (Table S4). In males, statistically significant decreases were observed in triglycerides, urea, BUN, creatinine, and potassium in certain dose groups when compared with the control (G1). In females, statistically significant decreases were noted in triglycerides and total bilirubin, whereas increases were observed in albumin, globulin, and total protein in some treated groups (G2–G4). A significant increase in calcium (G3 and G4R) and a significant decrease in ALP (G4) was also recorded.

Despite these statistical variations, all biochemical and electrolyte parameters remained within physiological and historical control ranges, and there was no consistent dose-related trend. These alterations lacked correlation with related organ-weight or histopathological findings, particularly in the liver and kidneys, which showed no treatment-related microscopic lesions. Furthermore, the changes did not persist in the recovery groups (G1R and G4R). Overall, the biochemical and electrolyte findings were considered to reflect normal biological variability rather than toxicologically meaningful or test item–related effects.

### Urinalysis

**Wistar rats.** There were no test-item–related changes observed in any urinalysis parameter. Urinary appearance, volume, pH, and specific gravity were comparable between treated groups (G2, G3, G4, and G4R) and their respective controls (G1 and G1R). Biochemical constituents—includi nirabolam, bilirubin, urobilinogen, ketones, and glucose-showed no statistically significant differences across groups. Microscopic examination of the urine sediment revealed no treatment-related alterations, with cast cells, crystals, and epithelial cells occurring at similar frequencies in both control and treated animals of either sex (Table 6).

These findings indicate that administration of *E. coli* 5C did not induce any adverse effects on renal function or urinary constituents in Wistar rats.

**Athymic nude mice.** Urinalysis was not conducted in nude mice, as this assessment was not considered necessary for the objectives of the non-GLP toxicity study.

### Gross Pathology

**Wistar rats.** No treatment-related gross pathological changes were observed in any animals. External and internal examinations at necropsy revealed no lesions attributable to the test item across all groups. Occasional findings (*e.g.*, unilateral ovarian cyst in one female) were considered spontaneous and incidental, as such changes occur naturally in laboratory rodents and showed no relationship to dose or treatment (Table 7).

**Athymic nude mice.** No treatment-related gross pathological changes were observed in any of the animals across the control or test item–treated groups. External examinations revealed no abnormalities in any dose group. Internal gross examinations similarly showed no test-item–related lesions in any organ (Table S5).

### Organ Weights

**Wistar rats.** Absolute organ weight data are presented in Table 8. No statistically significant differences were observed in the absolute weights of any major organs—including the brain, heart, adrenals, spleen, kidneys, liver, thymus, testes, epididymides, prostate with seminal vesicles and coagulating glands (males), uterus, and ovaries (females)—in the treated groups (G2, G3, G4, and G4R) when compared with their respective control groups (G1 and G1R). All values remained within normal biological variation, and no treatment-related effects on organ weights were identified.

**Athymic nude mice.** Absolute organ weights for both sexes are presented in Table S6. Across all treated groups (G2, G3, G4, and G4R), the absolute weights of major organs—including the brain, heart, adrenals, spleen, kidneys, liver, testes, epididymis, prostate with seminal vesicles/coagulating glands (males), uterus with cervix, and ovaries (females)—were generally comparable to those of the respective control groups (G1 and G1R). In males, statistically significant decreases were noted in the heart, kidneys, and liver in G2 when compared with the G1 control, and a statistically significant increase in spleen weight was observed in G3. In females, a statistically significant decrease in kidney weight was observed when compared with G1. However, all these variations were isolated, lacked dose-dependency, and did not correlate with any microscopic abnormalities. Overall, no treatment-related changes in absolute organ weights were identified in either sex.

### Histopathology

**Wistar rats.** Histopathological findings are summarized in Table 9. Histopathological examination was conducted on animals from the control (G1) and high-dose (G4) groups, with five males and five females evaluated in each group. No test-item–related microscopic lesions were observed in any examined organs. All findings were considered spontaneous, incidental, and consistent with background changes typically observed in Wistar rats.

The details of organs examined are presented in section 2.11 Across all these tissues, no abnormalities attributable to the test item were detected. Microscopic findings were comparable between treated and control groups and fell within expected biological variation, confirming that *E. coli* 5C induced no histopathological effects under the conditions of this study. Representative hematoxylin and eosin (H&E)–stained sections of the intestinal mucosa, mesenteric lymph nodes, liver, and spleen from both control and high-dose treated animals are provided in the Supplementary Material (Fig. S1). These images further support the absence of treatment-related histopathological alterations.

**Athymic nude mice.** Histopathological findings are summarized in Table S7. Examination was performed on animals from the control (G1) and high-dose (G4) groups, comprising five males and five females in each group. No test-item–related microscopic lesions were identified in any of the examined tissues. All observations were consistent with spontaneous background changes commonly reported in this strain of nude mice.

The details of organs examined are presented in section 2.11. Incidental findings such as minimal inflammatory cell infiltrates, multifocal congestion and hemorrhage in the lungs or kidneys, focal gastric hyperkeratosis, mild mononuclear infiltrates in the intestines, and occasional hypospermia or germ-cell exfoliation in the male reproductive organs were noted sporadically. These changes occurred with similar frequency in both control and treated groups, lacked dose-dependency, and bore no relationship to test item administration. Overall, histopathological evaluation demonstrated no microscopic evidence of toxicity attributable to *E. coli* 5C in male or female nude mice. Representative H&E–stained sections of the intestinal mucosa, mesenteric lymph nodes, liver, and spleen from both control and high-dose–treated animals are provided in the Supplementary Material (Fig. S2). These images further confirm the absence of treatment-related histopathological alterations and support the conclusion that *E. coli* 5C did not induce tissue injury or evidence of systemic dissemination under the conditions of the study.

### NOAEL Statement

Based on the results of the GLP-compliant 28-day repeated-dose oral toxicity study in Wistar rats and the parallel non-GLP study in athymic nude mice, *E. coli* 5C was well tolerated at all tested dose levels. No treatment-related adverse effects were observed in clinical signs, mortality, body weight, feed consumption, functional observations, hematology, clinical chemistry, urinalysis, organ weights, gross pathology, or histopathology in either species, at any dose level. Accordingly, the highest tested dose of 2,000 mg/kg/b.wt. (~2 × 10^11^ CFU/kg) was identified as the NOAEL for both male and female Wistar rats and athymic nude mice under the conditions of this study.

## Discussion

The safety assessment of probiotic microorganisms intended for human use requires a rigorous, multi-layered approach, especially when the candidate species belongs to *E. coli*, a bacterial species containing both benign commensals and potentially pathogenic variants. In the present work, we performed a comprehensive subacute toxicity evaluation of *E. coli* 5C, a newly characterized *pks*-negative strain isolated from the stool of a healthy infant and previously shown to be free of virulence factors, plasmids, and antimicrobial-resistance determinants [41]. Genomic analysis has further confirmed the strain’s absence of the *pks* island—a key determinant responsible for colibactin biosynthesis, mutagenicity, and tumor-promoting potential in certain *E. coli* lineages—and established the strain’s genetic stability, antibiotic sensitivity, and overall safety profile [42]. These genomic characteristics align with international principles for probiotic safety evaluation (Food and Agriculture Organization (FAO), World Health Organization (WHO) and the European Food Safety Authority’s (EFSA) Qualified Presumption of Safety (QPS)) framework, which emphasize the importance of demonstrating the absence of virulence genes, antimicrobial-resistance determinants, and genotoxicity-related loci.

The findings of our GLP-compliant 28-day repeated-dose oral toxicity study in healthy Wistar rats demonstrate that *E. coli* 5C was well tolerated at all tested dose levels, including the limit dose of 2,000 mg/kg b.wt. (~2 × 10^11^ CFU/kg). The absence of mortality, adverse clinical signs, or changes in body weight, feed consumption, FOB, hematological parameters, clinical biochemistry, urinalysis, organ weights, or gross and microscopic pathology indicates that daily oral exposure to extremely high microbial loads did not result in systemic toxicity or organ-specific adverse effects . Importantly, no evidence of microbial translocation was identified, as indicated by the absence of clinical signs of infection, normal hematological and biochemical profiles, and the lack of histopathological lesions in lymphoid and non-lymphoid organs. These findings support the conclusion that the observed minor statistical variations in some parameters were within historical reference ranges and represent incidental biological variability rather than toxicologically meaningful effects. This is a critical consideration in the safety evaluation of live microorganisms, as systemic dissemination represents a key theoretical risk associated with probiotic use. The absence of such findings supports the conclusion that the observed minor statistical variations in some haematological and biochemical parameters were within historical reference ranges and represent incidental biological variability rather than toxicologically meaningful effects.

The establishment of a clear NOAEL further strengthens the toxicological evaluation of *E. coli* 5C. To our knowledge, a subacute 28-day NOAEL has not previously been determined for an *E. coli* probiotic strain. In this study, no adverse effects were observed at any dose level, and the highest tested dose of 2000 mg/kg/ b.wt./day (~2 × 10^11^ CFU/kg/day) was therefore identified as the NOAEL in Wistar rats. Importantly, the absence of any adverse findings at the same dose in immunocompromised athymic nude mice provides supportive evidence that *E. coli* 5C is well tolerated even under conditions of reduced immune competence. This level of tolerability is notable when compared with reported NOAELs for other probiotic microorganisms. For example, a NOAEL of 5,000 mg/kg/day (≈4.71 × 10^9^ CFU/kg/day) was reported for *Lactobacillus curvatus* WiKim 38 (LCW) [53], while the EFSA-reviewed *Clostridium butyricum* TO-A strain demonstrated a NOAEL of 3,000 mg/kg/day (≈10^9^–10^10^ CFU/kg/day) [54]. When viewed in this context, the NOAEL established for *E. coli* 5C indicates a level of tolerability comparable to that of well-recognized probiotic species.

Given that probiotics are not only consumed by healthy individuals but may also be used in populations with compromised immunity, evaluating safety in an immunodeficient model is particularly relevant. Although probiotics are generally regarded as safe, several reviews have noted that immunocompromised hosts may theoretically be more susceptible to adverse events or infections from live microorganisms [55-59]. At the same time, probiotics are still sometimes recommended—for example, in newborns and infants, young children, elderly individuals, or patients receiving steroid therapy-groups in which varying degrees of immune immaturity or secondary immunosuppression may be present [60-63]. These considerations highlight the importance of assessing the safety of *E. coli* 5C under conditions of reduced immune competence. For this reason, we included athymic nude mice-an established model for impaired adaptive immunity-in the present study. The 28-day oral administration of *E. coli* 5C in athymic nude mice demonstrated favourable tolerability, with no mortality, no clinical or behavioral abnormalities, and no meaningful alterations in haematological, biochemical, or histopathological endpoints. Importantly, no evidence of microbial translocation or systemic infection was observed, as indicated by the absence of clinical signs, inflammatory or haematological abnormalities, and histopathological lesions in lymphoid, hepatic, or renal tissues. The lack of such findings in this hig*hly* sensitive immunodeficient model-where adverse effects would be expected to manifest if present—provides strong support for the safety of *E. coli* 5C under conservative, high-dose exposure conditions. The toxicological findings are consistent with the strain’s previously reported biological behavior. *In vitro* assays have shown that *E. coli* 5C displays strong survival in simulated gastric and intestinal conditions, efficient epithelial adhesion, acetate production, low histamine output, and beneficial immunomodulatory effects (IL-10, IL-12 induction) [41]. The convergence between these *in vitro* functional characteristics and the absence of adverse effects *in vivo* reinforces the biological plausibility that *E. coli* 5C represents a benign commensal strain. Additionally, preliminary clinical evidence suggests that *E. coli* 5C may accelerate recovery of the gut microbiota and alleviate gastrointestinal discomfort following colonoscopy, with rapid symptom improvement compared with historical experience of the same individuals [43]. Taken together, these toxicological, genomic, and functional findings consistently support the probiotic candidacy of *E. coli* 5C.

From a comparative standpoint, *E. coli* 5C appears to possess significant advantages over traditional *E. coli* probiotics, such as *E. coli* Nissle 1917, DSM 17252 and A0 34/86. These strains, although clinically used for decades, contain genetic elements of concern—including the *pks* island in *E. coli* Nissle 1917, and in DSM 17252, or *hly* and *cnf1* in the A0 34/86 strain—which may theoretically contribute to genotoxicity or severe inflammatory responses [40, 64]. Similar investigations conducted on *E. coli* Nissle 1917 demonstrated that its safety is dependent on the integrity of the host immune system, with studies showing that the strain can elicit toxicity in models with compromised adaptive immunity [65]. These observations further highlight the relevance of evaluating *E. coli* 5C in both immunocompetent and immunodeficient hosts, as performed in the present study. In contrast, *E. coli* 5C is potentially free of these genetic markers and appears to be among the few *E. coli* strains for which comprehensive genomic, functional, and toxicological safety data have been reported [41]. However, direct comparative studies would be needed to firmly establish these differences.

Overall, the current findings robustly demonstrate that repeated oral administration of *E. coli* 5C does not induce systemic, biochemical, or histopathological toxicity in either immunocompetent or immunocompromised rodent models. Together with existing genomic and functional evidence, these results support the continued clinical development of *E. coli* 5C as a probiotic candidate with a favorable safety profile. While the present study demonstrates short-term safety, further investigations—including chronic toxicity, reproductive and developmental toxicity, and carcinogenicity studies—are warranted to fully characterize the long-term safety of *E. coli* 5C, particularly in the context of prolonged or widespread human use.

## Conclusion

In conclusion, *Escherichia coli* 5C demonstrated a favourable safety profile following 28 days of repeated oral administration in both healthy Wistar rats and immunocompromised athymic nude mice. Across all evaluated endpoints—including clinical signs, behaviour, body weight, feed intake, haematology, biochemistry, organ weights, gross pathology, and histopathology—no treatment-related toxicity or organ-specific alterations were identified in either sex of either species. No treatment-related adverse effects were observed at any dose level, including the highest administered dose of 2,000 mg/kg/ b.wt./day (~2 × 10^11^ CFU/kg/day), which is therefore established as the NOAEL for both species under the conditions of this study.

The combined dataset from this toxicological evaluation, together with previously published genomic, mechanistic, and preliminary clinical findings, supports the view that *E. coli* 5C possesses a favourable safety profile as a potential probiotic strain. Its *pks*-negative genotype, absence of known virulence factors, antibiotic-sensitivity profile, and consistent lack of toxicity in both immunocompetent and immunocompromised models provide a strong foundation for further development. Importantly, the absence of treatment-related findings in both systemic and immune-deficient models suggests that *E. coli* 5C is well tolerated even under conservative, high-dose exposure conditions. Nevertheless, larger and well-controlled clinical studies remain necessary to further confirm its safety and to define its potential applications in diverse human populations.

Overall, the current evidence indicates that *E. coli* 5C may be suitable for continued clinical development as a probiotic candidate aimed at supporting, restoring, and modulating the gut microbiota.

## Supplemental Materials

Supplementary data for this paper are available on-line only at http://jmb.or.kr.



## Figures and Tables

**Table 1:** Table 1

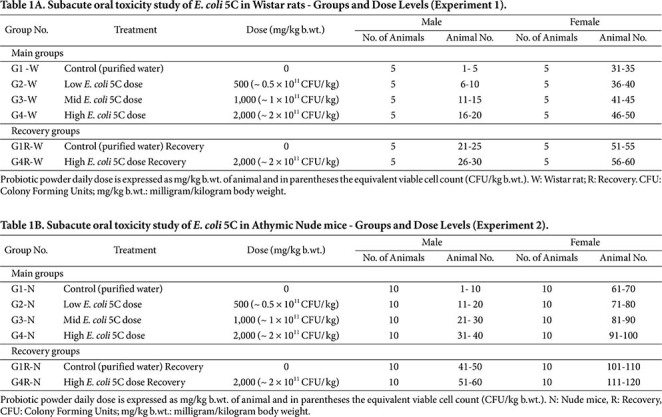

**Table 2:** Subacute oral toxicity study of *E. coli* 5C in Wistar rats - Weekly Body weight Values (Main and Recovery groups).

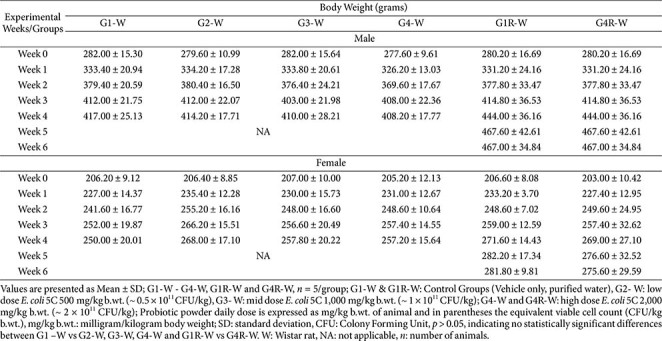

**Table 3:** Subacute oral toxicity study of *E. coli* 5C in Wistar rats - Motor Activity and Grip Strength (Main & Recovery Groups).

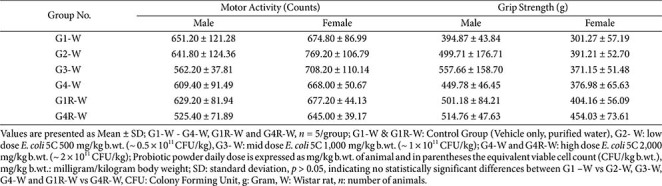

**Table 4:** Table 4

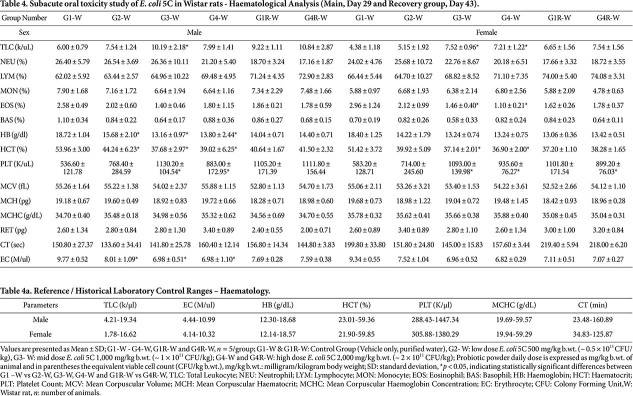

**Table 5:** Table 5

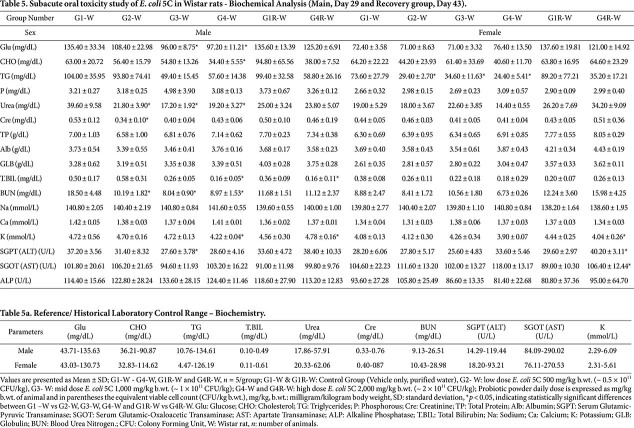

**Table 6:** Subacute oral toxicity study of *E. coli* 5C in Wistar rats - Urinary Parameters (Main, Week 4 and Recovery group, Week 6).

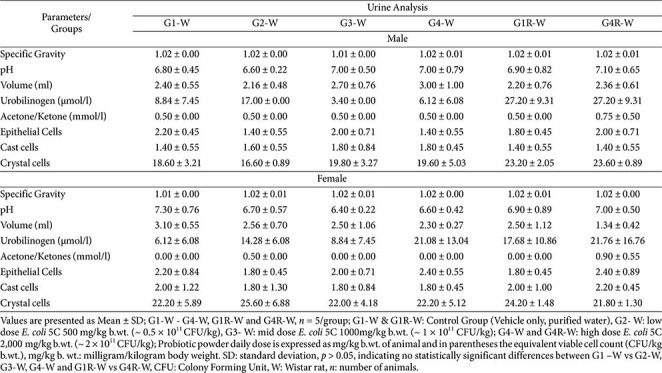

**Table 7:** Subacute oral toxicity study of *E. coli* 5C in Wistar rats - Summary of Gross pathology (Main, Day 29 and Recovery group, day 43).

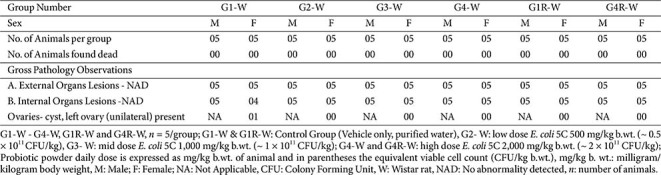

**Table 8:** Subacute oral toxicity study of *E. coli* 5C in Wistar rats - Absolute Organ Weight (Main, Day 29 and Recovery group, Day 43).

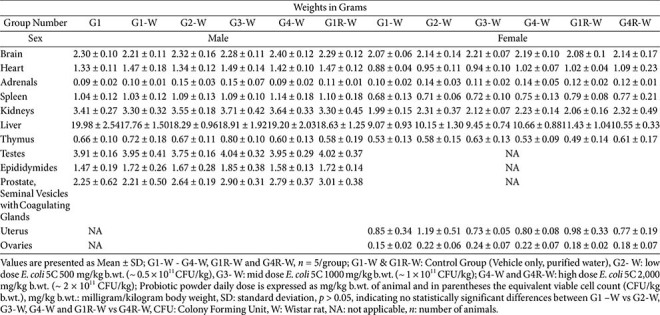

**Table 9:** Subacute oral toxicity study of *E. coli* 5C in Wistar rats - Summary of Histopathology (Main groups).