# Supplementary material for: Subacute 28-Day OECD-Guided Oral Toxicity Study of Escherichia coli 5C (LMG S-33222) in Wistar Rats and Immunocompromised Nude Mice
Source: J Microbiol Biotechnol. 2026 Jan 26;36:e2512003. doi: 10.4014/jmb.2512.12003 (PMC12868953; doi:10.4014/jmb.2512.12003)
Supplement: Supplementary file 1 [file jmb-36-e2512003-supple.pdf]

Supplementary Table 1: Subacute oral toxicity study of *E. coli* 5C in Athymic Nude Mice - Weekly Body weight Values (Main and Recovery groups).

Experimental Weeks/Groups	Body Weight (grams)					
	**G1-N**	**G2-N**	**G3-N**	**G4-N**	**G1R-N**	**G4R-N**
	**Male**					
Week 0	29.31±1.43	26.44±2.36	29.22±2.21	28.5±1.94	28.87±1.78	28.33±1.99
Week 1	28.3±1.52	25.62*±2.18	28.35±2.27	28.21±1.5	27±2.83	26.33±2.01
Week 2	27.79±1.79	25.85±2.05	27.62±2.13	27.27±1.83	28.14±1.67	26.72±1.98
Week 3	28.28±1.73	27.7±2.65	28.54±2.00	27.03±1.8	29.01±1.77	27.1±2.36
Week 4	29.17±1.8	26.98±2.56	29.11±2.25	27.88±2.2	29.6±2.04	28±2.05
Week 5	NA				29.83±2.21	28.67±2.35
Week 6					30.37±2.37	29.78±2.58
**Female**						
Week 0	25.39±0.78	25.85±1.82	24.98±1.28	25.63±.12	24.48±1.07	26.25±1.71
Week 1	24.08±1.18	23.99±1.43	23.37±2.53	24.31±2.46	23.96±1.33	24.32±2.44
Week 2	24.07±0.78	24.1±1.87	24.9±1.86	24.54±1.74	23.96±1.26	25.49±2.16
Week 3	24.95±1.08	25.18±1.96	25.09±1.97	24.45±2.17	25.03±1.13	26.43*±1.69
Week 4	25.34±0.94	25.48±2.24	25.7±2.28	24.4±3.09	25.59±1.16	26.6±1.31
Week 5	NA				26.04±1.17	27.19±1.88
Week 6					26.53±1.18	27.88±2.23

NOTE: Values are presented as Mean ± SD; G1-N - G4N, G1R-N and G4R-N, *n*=10/group; G1-N & G1R-N: Control Group (Vehicle only, purified water), G2- N: low dose *E. coli* 5C 500 mg/kg b.wt. (~ 0.5 × 10^{11} CFU/kg), G3-N: mid dose *E. coli* 5C 1000 mg/kg b.wt. (~ 1 × 10^{11} CFU/kg); G4-N and G1R-N: high dose *E. coli* 5C 2000 mg/kg b.wt. (~ 2 × 10^{11} CFU/kg); Probiotic powder daily dose is expressed as mg/kg b.wt. of animal and in parentheses the equivalent viable cell count (CFU/kg b.wt.), mg/kg b. wt.: milligram/kilogram body weight, SD: standard deviation, *$p < 0.05$, indicating statistically significant differences between G1-N vs G2-N, G3-N, G4-N and G1R-N vs G4R-N. N: Nude mice, CFU: Colony Forming Unit, NA: not applicable, *n*: number of animals.

Supplementary Table 2: Subacute oral toxicity study of *E. coli* 5C in Athymic Nude Mice – Motor Activity in Open Field test.

Group No.	Crossings (No.)	Rearing (No.)	Grooming (No.)	Time in Periphery (sec)	Time in Centre (sec)	Entries into Centre (No.)
**Male**						
G1-N	28.80±3.19	3.60±0.55	2.80±1.30	167.00±1.58	13.00±1.58	13.40±4.39
G2-N	25.40±3.65	3.80±0.84	2.40±0.55	164.80±5.45	15.20±5.45	14.00±2.24
G3-N	27.40±5.18	3.60±1.14	3.00±0.71	165.60±4.10	14.40±4.10	15.20±2.39
G4-N	27.80±3.27	3.80±0.45	3.20±1.10	166.20±3.11	13.80±3.11	15.80±5.07
G1R-N	23.60±2.41	5.00±0.71	5.60±1.14	165.60±2.70	14.40±2.70	16.40±3.85
G4R-N	28.60*±4.16	3.40*±1.14	5.40±0.89	166.00±2.55	14.00±2.55	20.40±3.05
**Female**						
G1-N	27.2±4.09	3.8±1.48	2.8±0.84	168.2±4.27	11.8±4.27	11.2±3.96
G2-N	25.4±3.21	3.4±0.55	3.0±1.0	164.8±3.56	15.2±3.56	13.8±2.39
G3-N	28±3.61	5.6±1.34	3.4±0.89	167.6±5.32	12.4±5.32	16.4±2.41
G4-N	25.6±4.22	3.8±1.92	3.4±0.55	165.4±3.51	14.6±3.51	20.00*±6.24
G1R-N	25.4±4.51	3.4±0.89	3.6±1.52	167.6±2.88	12.4±2.88	18.8±4.02
G4R-N	23.2±2.59	4.2±0.45	3.6±2.07	165.4±3.65	14.6±3.65	18.6±4.16

NOTE: Values are presented as Mean ± SD; G1-N - G4N, G1R-N and G4R-N, *n*=10/group; G1-N & G1R-N: Control Group (Vehicle only, purified water), G2- N: low dose *E. coli* 5C 500 mg/kg b.wt. (~ 0.5 × 10^{11} CFU/kg), G3-N: mid dose *E. coli* 5C 1000 mg/kg b.wt. (~ 1 × 10^{11} CFU/kg); G4-N and G1R-N: high dose *E. coli* 5C 2000 mg/kg b.wt. (~ 2 × 10^{11} CFU/kg); Probiotic powder daily dose is expressed as mg/kg b.wt. of animal and in parentheses the equivalent viable cell count (CFU/kg b.wt.), mg/kg b. wt.: milligram/kilogram body weight, SD: standard deviation, *$p < 0.05$, indicating statistically significant differences between G1-N vs G2-N, G3-N, G4-N and G1R-N vs G4R-N. CFU: Colony Forming Unit, N: Nude, mice, *n*: number of animals.

Supplementary Table 3: Subacute oral toxicity study of *E. coli* 5C in Athymic Nude Mice - Haematological Analysis (Main, Day 29 and Recovery group, Day 43).

Group Number	G1-N	G2-N	G3-N	G4-N	G1R-N	G4R-N	G1-N	G2-N	G3-N	G4-N	G1R-N	G4R-N
**Sex**	**Male**						**Female**					
WBC 10^3/µL	3.18±1.12	2.4±1.61	1.95±0.24	1.92±0.8	2.58±0.81	1.92±0.71	1.3±0.28	1.92±0.75	1.73±0.78	1.56±0.77	2.57±1.42	3.24±1.86
NEU (%)	29.88±4.48	36.86±6.61	28.7±11.78	30.16±5.95	47.5±5.03	48.44±12.37	33.3±7.95	40.14±6.39	30.76±4.21	41.5±9.42	44.36±11.76	35.88±15.63
LYM (%)	64.62±3.58	59.36±6.45	67.74±11.37	66.72±5.6	47.22±6.32	46.44±10.07	62.56±8	56.24±7.54	65.24±4.53	52.24±5.36	51.42±12.23	58.88±13.87
MON (%)	3.7±1.73	2.08±0.77	1.78±0.77	1.92±1.48	2.18±67	1.38±0.33	1.4±1.39	1.3±1.47	1.38±0.72	2.88±1.75	1.28±1.04	1.92±1.76
EOS (%)	1.54±0.94	1.42±0.61	1.48±0.43	0.92±0.54	2±1.37	2.56±2.28	2.18±2.03	1.58±0.89	2.24±1.29	1.92±1.75	1.04±0.38	2.88±3.53
BAS (%)	0.26±0.17	0.28±0.27	0.3±0.17	0.28±0.19	1.1±0.52	1.18±0.68	0.56±0.36	0.74±0.15	0.38±0.11	1.46±1.21	1.9±0.57	0.44*±0.19
RBC10^6/µL	6.16±1.73	6.08±2.12	5.77±0.62	5.12±0.77	8.63±2.33	5.96*±0.59	5.45±1.05	5.26±1.09	5.86±1.08	5.69±1.23	5.81±0.36	6.7±1.86
HB (g/dl)	9.36±2.35	9.3±3.32	8.82±0.84	8.04±1.16	13.36±3.48	9.18*±0.89	8.64±1.62	8.98±1.18	9.08±1.68	9.02±1.87	9±0.42	10.42±2.88
HCT (%)	27.08±7.26	27±9.88	25.56±2.59	22.98±3.59	38.66±9.9	26.88*±2.57	24.24±4.73	25.46±3.31	25.9±4.67	25.1±5.49	25.38±1.19	30.1±8.19
PLT (K/uL)	794.4±189.04	563.6±87.67	607.4±167.73	572.6±130.88	550.4±285.13	830.6±117.72	471.4±156.98	560.6±129.36	618.4±205.99	500.4±177.87	736.2±90.33	464.20*±144.79
MCV (fL)	44.06±0.89	44.26±1.16	44.28±1.31	44.92±1.23	45±1.21	45.14±0.64	44.48±0.65	44.58±1.72	44.24±1.33	44.16±1.16	43.76±1.97	44.96±1.52
MCH (pg)	15.28±0.6	15.24±0.42	15.36±0.43	15.74±0.33	15.54±0.36	15.38±0.28	15.88±0.33	15.7±0.49	15.54±0.29	15.84±0.21	15.46±0.49	15.54±0.48
MCHC (g/dL)	34.68±0.88	34.44±0.69	34.68±0.65	35.02±0.63	34.46±0.64	34.1±0.2	35.7±0.48	35.26±0.57	35.14±0.47	35.94±1.12	35.38±1.44	34.56±0.51
RT%	3.2±1.3	3.4±1.14	4.4±1.52	3.6±1.14	3.2±0.84	3.2±1.1	4.4±1.52	4±0.71	3.4±1.34	3±1.58	3±1	3.2±0.84
CT (sec)	72±14.09	90.80*±7.73	71.8±6.3	166.40*±13.46	237.2±4.09	226.80*±5.54	83.8±5.02	129.40*±7.13	141.40*±11.5	119.2±47.37	219.4±5.94	218±6.2

NOTE: Values are presented as Mean ± SD; G1-N - G4-N, G1R-N and G4R-N, *n*=10/group; G1-N & G1R-N: Control Group (Vehicle only, purified water), G2- N: low dose *E. coli* 5C 500 mg/kg b.wt. (~ 0.5 × 10^{11} CFU/kg), G3-N: mid dose *E. coli* 5C 1000 mg/kg b.wt. (~ 1 × 10^{11} CFU/kg); G4-N and G1R-N: high dose *E. coli* 5C 2000 mg/kg b.wt. (~ 2 × 10^{11} CFU/kg); Probiotic powder daily dose is expressed as mg/kg b.wt. of animal and in parentheses the equivalent viable cell count (CFU/kg b.wt.), mg/kg b.wt.: milligram/kilogram body weight, SD: standard deviation, *$p < 0.05$, indicating statistically significant differences between G1-N vs G2-N, G3-N, G4-N and G1R-N vs G4R-N. N: Nude mice; TLC: Total Leukocyte; NEU: Neutrophil; LYM: Lymphocyte; MON: Monocyte; EOS: Eosinophil; BAS: Basophil; HB: Haemoglobin; HCT: Haematocrit; PLT: Platelet Count; MCV: Mean Corpuscular Volume; MCH: Mean Corpuscular Hematocrit; MCHC: Mean Corpuscular Haemoglobin Concentration; EC: Erythrocyte, CFU: Colony Forming Unit, *n*: number of animals.

Supplementary Table 4: Subacute oral toxicity study of *E. coli* 5C in Athymic Nude Mice - Biochemical Analysis (Main, Day 29 and Recovery group, Day 43).

Group Number	G1-N	G2-N	G3-N	G4-N	G1R-N	G4R-N	G1-N	G2-N	G3-N	G4-N	G1R-N	G4R-N
Sex	Male						Female					
Glu (mg/dL)	99.37±32.87	87.68±9.8	97.86±8.84	89.01±35.06	160.38±37.46	183.44±41.51	73.36±16.13	62.3±12.43	74.29±30.58	88.28±37.91	161.84±24.71	186.78±24.06
CHO (mg/dL)	77.6±20.26	96.7±33.9	77.73±45.47	77.28±22.02	119.15±15.48	139.22±41.43	37.98±14.28	122.35*±39.67	116.92*±43.58	89.63±21.61	112.38±12.12	115.01±19.14
TG (mg/dL)	64.79±10.82	81.3±42.8	65.06±17.2	60.86±13.64	81.55±63.54	90.3±29.37	43.51±7.38	54.12±18.28	76.5±37.71	19.48±9.39	126.66±18.25	87.34±38.94
T.BIL (mg/dL)	0.66±0.05	0.12*±0.12	0.42*±0.17	0.16*±0.17	1.24±0.24	0.97±0.31	0.22±0.17	0.07±0.06	0.3±0.09	0.29±0.27	0.93±0.39	0.87±0.2
P (mg/dL)	6.43±0.68	7.27±.88	5.7±1.54	5.6±2.3	8.32±2.93	6.44±1.84	6.47±0.35	10.5±4.82	12.34*±2.46	11.43*±4.49	6.15±1.68	6.6±1.47
Urea (mg/dL)	52.22±25	65.27±18.74	58.75±17.64	57.56±8.16	38.12±12.53	44.31±15.45	67.89±18.16	59.35±12.69	54.38±9.92	83±39.64	32.05±12.05	24.19±10.19
BUN (mg/dL)	24.4±11.68	30.5±8.76	27.45±8.24	26.9±3.81	17.81±5.85	20.7±7.22	31.73±8.49	27.73±5.93	25.41±4.64	38.79±18.53	14.98±5.63	11.31±4.76
GLB (g/dL)	2.15±0.98	1.84±1.15	3.42±5.13	10.23*±1.79	3.67±1.34	4.21±1.01	1.25±0.72	5.41±5.35	3.3±3.87	10.66*±1.02	4.09±0.65	3.35±1.3
Cre (mg/dL)	0.43±0.19	0.41±0.1	0.3±0.09	0.48±0.06	0.22±0.03	0.33±0.2	0.56±0.54	0.46±0.09	0.37±0.11	0.34±0.13	0.21±0.01	0.41±0.35
TP (g/dL)	4.49±0.67	4.24±0.75	5.35±5.28	14.79*±0.63	5.31±1.23	6.27±1.19	3.75±0.72	7.82±5.43	5.45±4.46	15.46*±0.26	5.94±0.26	5.52±1.16
Alb (g/dL)	2.35±0.43	2.41±0.47	1.94±0.44	4.56*±1.73	1.64±0.48	2.06±0.26	2.5±0.18	2.41±0.09	2.15±0.68	4.80*±0.84	1.85±0.54	2.17±0.29
SGPT (ALT) (U/L)	15.49±9.15	42.25±25.85	24.45±16.01	21.88±12.79	22.23±4.69	17.97±4.55	30.72±16.38	32.34±20.53	41.7±26.98	30.98±26.22	29.03±20.93	17.79±4.07
SGOT (AST) (U/L)	51.6±4.22	40.8±20.71	51.6±11.17	52.4±25.83	27.4±2.51	36.2±9.31	61.4±10.92	64.4±20.46	38±11.25	83.6±18.8	35±8.15	34.6±3.65
ALP (U/L)	275.04±98.73	261.32±72.98	182.22±35.96	154.54*±24.04	137.49±77.01	115.84±12.08	340.78±98.45	284±77.99	242.48±56.92	138.38*±24.55	134.88±37.08	119.2±71.73
Na (mmol/L)	153.8±3.03	154.4±0.55	154.4±3.65	153.6±3.21	149.4±1.52	148.2±2.28	152.6±2.3	151.8±1.3	152.2±2.05	149.4±2.07	147.8±1.48	172.2±24.39
Ca (mmol/L)	1.11±0.03	1.12±0.02	1.16±0.03	1.16±0.03	1.18±0.03	1.22*±0.01	1.14±0.03	1.13±0.01	1.18*±0.01	1.16±0.03	1.25±0.05	0.46*±0.4
K (mmol/L)	6.14±0.68	6.92±0.59	7.08±1.02	7.28±1.31	5.36±0.42	6.12±0.27	6.12±0.61	5.44±0.24	6.3±0.44	8.28±3.52	5.38±0.44	5.92±0.82

NOTE: Values are presented as Mean ± SD; G1-N - G4-N, G1R-N and G4R-N, *n*=10/group; G1-N & G1R-N: Control Group (Vehicle only, purified water), G2- N: low dose *E. coli* 5C 500 mg/kg b.wt. (~ 0.5×10^{11} CFU/kg), G3-N: mid dose *E. coli* 5C 1000 mg/kg b.wt. (~ 1×10^{11} CFU/kg), G4-N and G1R-N: high dose *E. coli* 5C 2000 mg/kg. b.wt. (~ 2×10^{11} CFU/kg); Probiotic powder daily dose is expressed as mg/kg b.wt. of animal and in parentheses the equivalent viable cell count (CFU/kg b.wt.), mg/kg b.wt.: milligram/kilogram body weight, SD: standard deviation, *$p < 0.05$, indicating statistically significant differences between G1-N vs G2-N, G3-N, G4-N and G1R-N vs G4R-N. N: Nude mice;. Glu: Glucose; CHO: Cholesterol; TG: Triglycerides; P: Phosphorous; Cre: Creatinine; TP: Total Protein; Alb: Albumin; SGPT: Serum Glutamic-Pyruvic Transaminase; SGOT: Serum Glutamic-Oxaloacetic Transaminase; AST: Apartate Transaminase; ALP: Alkaline Phosphatase; T.BIL: Total Bilirubin; Na: Sodium; Ca: Calcium; K: Potassium; GLB: Globulin; BUN: Blood Urea Nitrogen, CFU: Colony Forming Unit, *n*: number of animals.

Supplementary Table 5: Subacute oral toxicity study of *E. coli* 5C in Athymic Nude Mice - Summary of Gross pathology (Main, Day 29 and Recovery group, Day 43).

Group Number	G1-N		G2-N		G3-N		G4-N		G1R-N		G4R-N	
Sex	M	F	M	F	M	F	M	F	M	F	M	F
No. of Animals per group	10	10	10	10	10	10	10	10	10	10	10	10
No. of animals found dead	00	00	00	00	00	00	00	00	00	00	00	00
Gross Pathology Observations												
A. External Lesions NAD	10	10	10	09	10	10	10	10	10	10	10	10
B. Internal Lesions -NAD	10	10	10	10	10	10	10	10	10	10	10	10

Note:Values are presented as Mean ± SD; G1-N - G4-N, G1R-N and G4R-N, *n*=10/group; G1-N & G1R-N: Control Group (Vehicle only, purified water), G2- N: low dose *E. coli* 5C 500 mg/kg b.wt. (~ 0.5×10^{11} CFU/kg), G3-N: mid dose *E. coli* 5C 1000 mg/kg b.wt. (~ 1×10^{11} CFU/kg); G4-N and G1R-N: high dose *E. coli* 5C 2000 mg/kg b.wt. (~ 2×10^{11} CFU/kg); Probiotic powder daily dose is expressed as mg/kg b.wt. of animal and in parentheses the equivalent viable cell count (CFU/kg b.wt.), mg/kg b.wt.: milligram/kilogram body weight, SD: standard deviation, N: Nude mice; M: Male, F: Female, CFU: Colony Forming Unit, NAD: No abnormality detected, *n*: number of animals.

Supplementary Table 6: Subacute oral toxicity study of *E. coli* 5C in Athymic Nude Mice - Absolute Organ Weight (Main, Day 29 and Recovery group, Day 43).

Weights (grams)												
**Group Number**	**G1-N**	**G2-N**	**G3-N**	**G4-N**	**G1R-N**	**G4R-N**	**G1-N**	**G2-N**	**G3-N**	**G4-N**	**G1R-N**	**G4R-N**
**Sex**	**Male**						**Female**					
Brain	0.52±0.05	0.49±0.06	0.52±0.06	0.53±0.05	0.5±0.04	0.51±0.06	0.52±0.03	0.55±0.04	0.52±0.03	0.49±0.02	0.51±0.03	0.53±0.05
Heart	0.17±0.02	0.15*±0.01	0.17±0.02	0.17±0.02	0.17±0.02	0.17±0.02	0.16±0.02	0.16±0.02	0.16±0.03	0.15±0.02	0.16±0.02	0.16±0.03
Adrenals	0.03±0.01	0.02±0.01	0.03±0.01	0.02±0.01	0.03±0.01	0.02*±0.01	0.03±0.01	0.03±0.01	0.02±0.00	0.02±0.01	0.03±0.01	0.03±0.01
Spleen	0.11±0.01	0.12±0.03	0.14±0.02	0.1±0.03	0.11±0.03	0.11±0.01	0.11±0.02	0.13±0.02	0.12±0.03	0.13±0.02	0.13±0.04	0.13±0.03
Kidneys	0.62±0.08	0.53*±0.06	0.58±0.08	0.56±0.07	0.61±0.06	0.55*±.05	0.48±0.07	0.45±0.07	0.43±0.04	0.4±0.04	0.49±0.04	0.46±0.05
Liver	1.81±0.17	1.53*±0.3	1.73±0.25	1.57±0.22	1.72±0.3	1.71±0.2	1.42±0.2	1.58±0.15	1.5±0.2	1.4±0.24	1.61±0.16	1.64±0.26
Testes	0.22±0.06	0.22±0.03	0.23±0.04	0.26±0.14	0.23±0.03	0.27±0.14	NA					
Epididymides	0.18±0.11	0.13±0.02	0.14±0.03	0.13±0.04	0.16±0.05	0.13±0.03	NA					
Prostate, Seminal Vesicles with Coagulating Glands	0.42±0.13	0.33±.11	0.33± 0.09	0.35±0.13	0.4±0.09	0.4±0.06	NA					
Uterus with cervix	NA						0.17±0.09	0.19±0.1	0.16±0.07	0.16±0.07	0.21±0.09	0.23±0.09
Ovaries	NA						0.06±0.06	0.05±0.02	0.05±0.03	0.05±0.01	0.06±0.02	0.03*±0.02

NOTE: Values are presented as Mean ± SD; G1-N - G4-N, G1R-N and G4R-N, *n*=10/group; G1-N & G1R-N: Control Group (Vehicle only, purified water), G2-N: low dose *E. coli* 5C 500 mg/kg b.wt. (~ 0.5 × 10^{11} CFU/kg), G3-N: mid dose *E. coli* 5C 1000 mg/kg b.wt. (~ 1 × 10^{11} CFU/kg); G4-N and G1R-N: high dose *E. coli* 5C 2000 mg/kg b.wt. (~ 2 × 10^{11} CFU/kg); Probiotic powder daily dose is expressed as mg/kg b.wt. of animal and in parentheses the equivalent viable cell count (CFU/kg b.wt.), mg/kg, b.wt.: milligram/kilogram body weight, SD: standard deviation, *$p < 0.05$, indicating statistically significant differences between G1-N vs G2-N, G3-N, G4-N and G1R-N vs G4R-N. N: Nude mice, NA: not applicable, CFU: Colony Forming Unit, *n*: number of animals.

Supplementary Table 7: Subacute oral toxicity study of *E. coli* 5C in Athymic Nude Mice **-** Summary of Histopathology. (Main groups).

S. No	Organ Name	Group Number	G1-N		G4-N	
		**Number of Animals**	**5**	**5**	**5**	**5**
		**Findings/ Sex**	**M**	**F**	**M**	**F**
1.	Gross lesion of external orifices	NAD	5	5	5	5
2.	Skin with Mammary gland	NAD	5	5	5	5
3.	Submandibular and Mesenteric lymph nodes	NAD	4	5	5	5
		Extramedullary hematopoiesis, megakaryocytes, multifocal, minimal	1	-	-	-
4.	Eyes	NAD	5	5	4	5
		Infiltration, inflammatory cells (Polymorphonuclear cells), retrobulbar space, unilateral, multifocal, mild	-	-	1	-
5.	Brain	NAD	5	5	5	5
6.	Thyroid gland	NAD	5	5	5	5
7.	Heart with Aorta	NAD	5	5	5	5
8.	Trachea	NAD	5	5	4	4
		Infiltration, inflammatory cells (mononuclear), focal, minimal	-	-	1	-
		Infiltration, inflammatory cells (mixed), multi focal, minimal	-	-	-	1
9.	Lungs	NAD	1	4	2	3
		Congestion and hemorrhage, multifocal, mild	4	1	3	2
10.	Stomach	NAD	3	4	4	4
		Infiltration, inflammatory cells (Mononuclear), focal, minimal	1	-	-	-
		Hyperkeratosis, orthokeratotic, multifocal, mild	1	1	1	1
11.	Small and large intestine (with payer's patches)	NAD	5	5	5	5
12.	Spleen and Adrenals	NAD	5	5	5	5
13.	Liver	NAD	5	5	4	5
		Infiltration, inflammatory cells (mononuclear), multifocal, minimal	-	-	1	-
14.	Kidneys	NAD	1	0	0	0
		Infiltration, inflammatory cells (mononuclear), multifocal, minimal	2	2	3	-
		Congestion and hemorrhage, multifocal, minimal	2	3	5	5
15.	Urinary bladder	NAD	5	5	5	5
16.	Testes, Epididymides and Male sex glands (Prostate, Seminal vesicles with coagulating glands)	NAD	5	NA	5	NA
17.	Ovaries, Uterus and Vagina	NAD	NA	5	NA	5
18.	Peripheral nerve (Sciatic nerve)	NAD	5	5	5	5
19.	Skeletal muscle	NAD	5	5	5	5
20.	Pituitary gland	NAD	5	5	5	5
21.	Femur bone with bone marrow	NAD	5	5	5	5
22..	Spinal cord	NAD	5	5	5	5

Note: NAD: No abnormality detected, NA: Not applicable, M: Male, F: Female, G1-N: Control (Vehicle only, purified water), G4- N: high dose *E. coli* 5C 2000 mg/kg b.wt. (~ 2 × 10^{11} CFU/kg); Probiotic powder daily dose

is expressed as mg/kg b.wt. of animal and in parentheses the equivalent viable cell count (CFU/kg b.wt.), mg/kg b.wt.: milligram/kilogram body weight;; N: Nude mice. Values shown indicate the number of animals in which the finding was observed, out of the number of animals examined microscopically in each group (*n*=5), *n*: number of animals.

<table>
<tr><th colspan="2">Supplementary Figure 1: Representative Histopathology images- H&E.X200 –Wistar rats</th></tr>
<tr><td>Group 1 Male - Spleen tissue</td><td>Group 1 Female - Spleen tissue</td></tr>
<tr><td>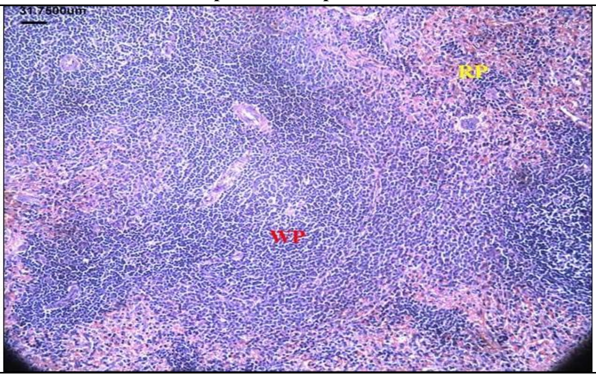
</td><td>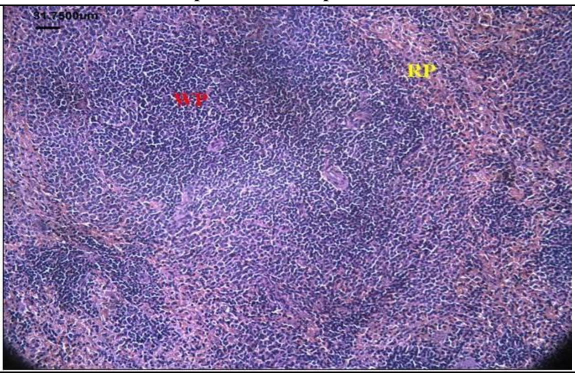
</td></tr>
<tr><td>Showing normal architecture of red pulp (RP) and white pulp (WP)</td><td>Showing normal architecture of red pulp (RP) and white pulp (WP)</td></tr>
<tr><td>Group 4 Male - Spleen tissue</td><td>Group 4 Female - Spleen tissue</td></tr>
<tr><td>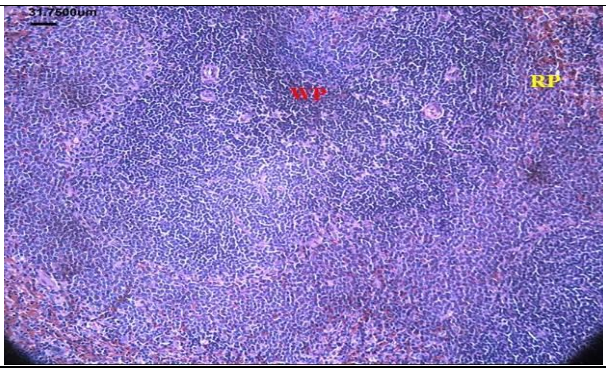
</td><td>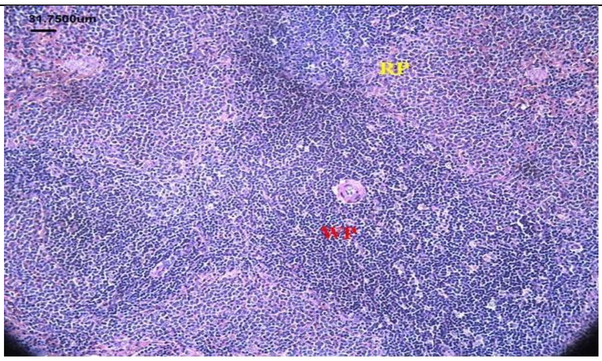
</td></tr>
<tr><td>Showing normal architecture of red pulp (RP) and white pulp (WP)</td><td>Showing normal architecture of red pulp (RP) and white pulp (WP)</td></tr>
<tr><td>Group 1 Male - Intestine</td><td>Group 1 Female - Intestine</td></tr>
<tr><td>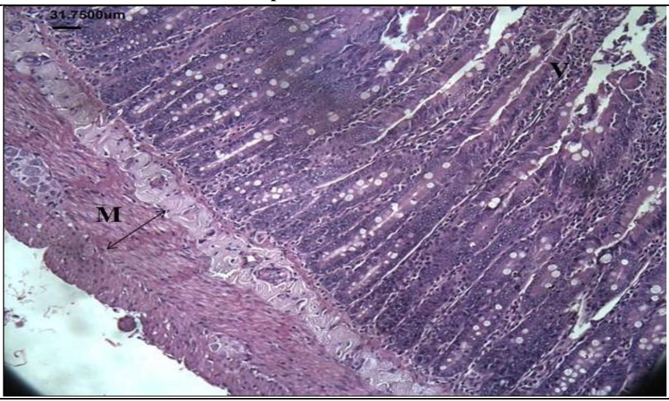
</td><td>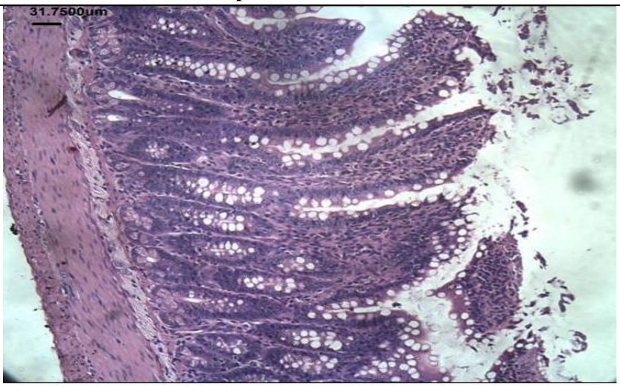
</td></tr>
<tr><td>Showing normal architecture of intestinal muscularis (M) and intestinal villi (V).</td><td>Showing normal architecture of intestinal muscularis (M) and intestinal villi (V).</td></tr>
<tr><td>Group 4 Male - Intestine</td><td>Group 4 Female - Intestine</td></tr>
<tr><td>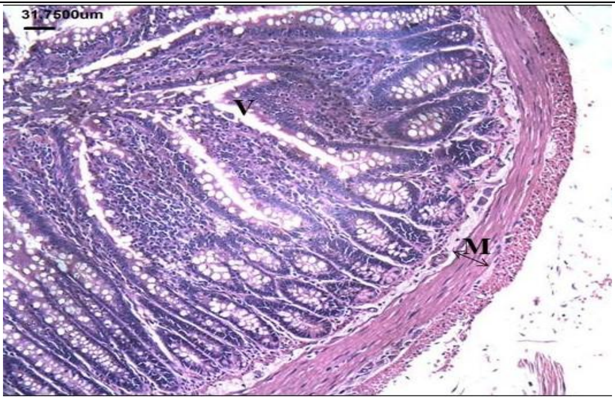
</td><td>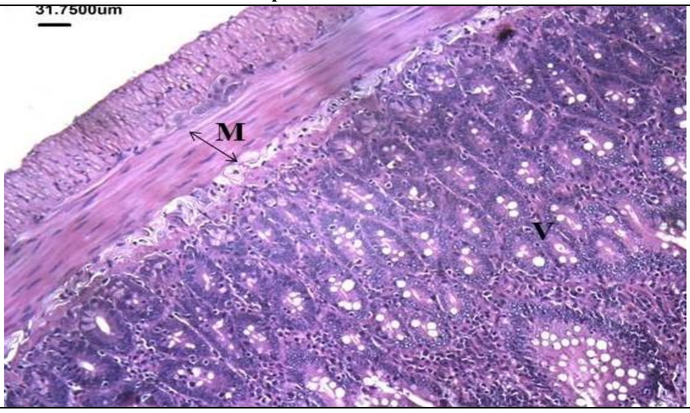
</td></tr>
<tr><td>Showing normal architecture of intestinal muscularis (M) and intestinal villi (V).</td><td>Showing normal architecture of intestinal muscularis (M) and intestinal villi (V).</td></tr>
</table>

**Supplementary Figure 1:** Representative Histopathology images- H&E.X200 –Wistar rats	
**Group 1 Male - Mesenteric lymph node**	**Group 1 Female - Mesenteric lymph node**
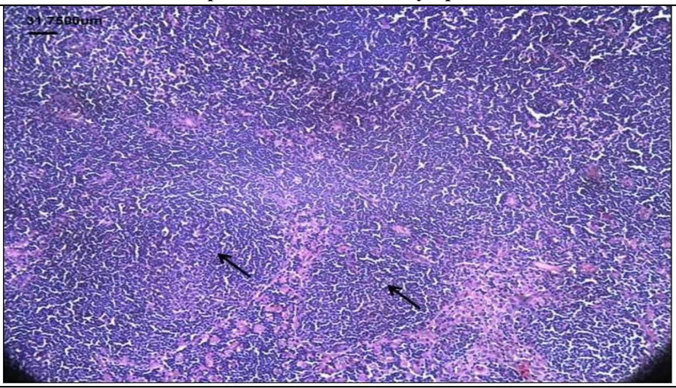	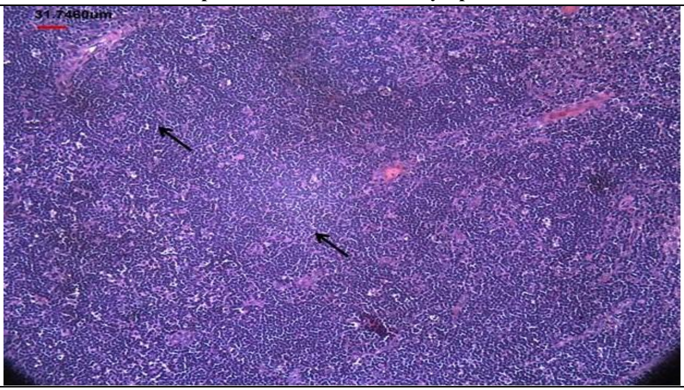
**Showing normal architecture of lymphoid follicle (arrow).**	**Showing normal architecture of lymphoid follicle (arrow).**
**Group 4 Male - Mesenteric lymph node**	**Group 4 Female - Mesenteric lymph node**
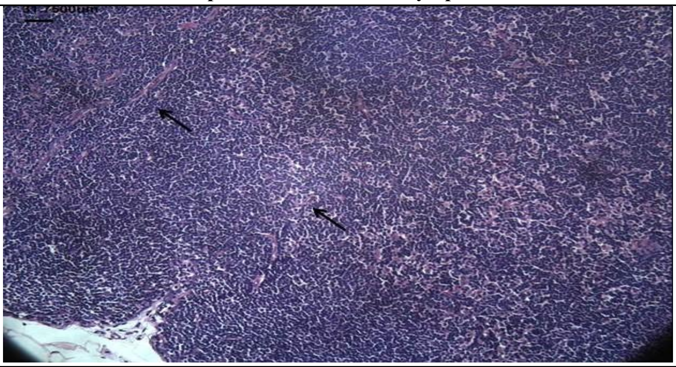	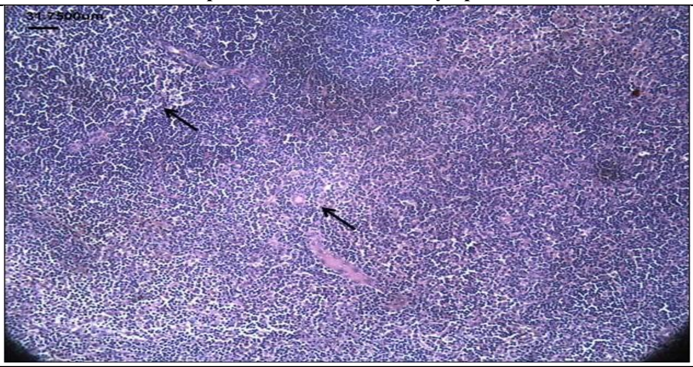
**Showing normal architecture of lymphoid follicle (arrow).**	**Showing normal architecture of lymphoid follicle (arrow).**
**Group 1 Male- Liver**	**Group 1 Female- Liver**
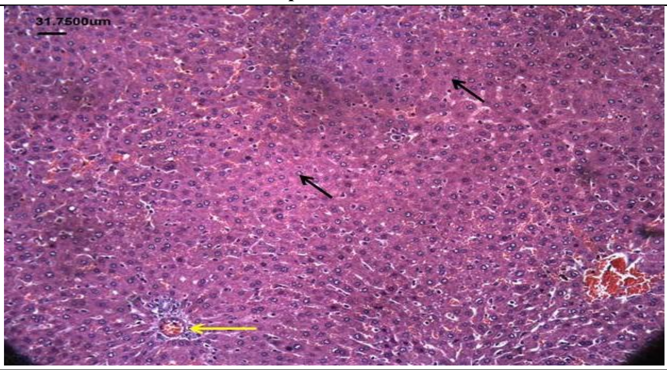	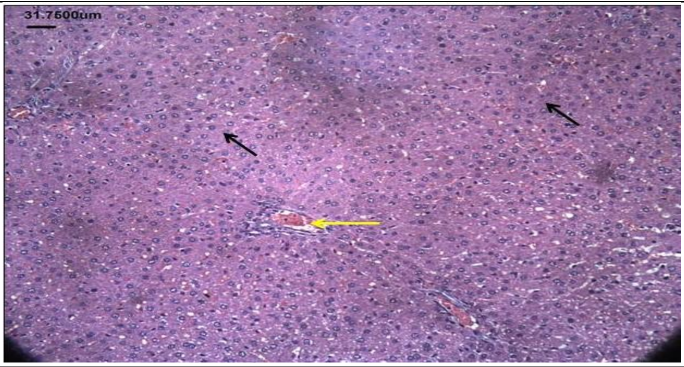
**Showing normal architecture of hepatocytes (arrow) with minimal congestion (yellow arrow).**	**Showing normal architecture of hepatocytes (arrow) with minimal congestion (yellow arrow).**
**Group 4 Male- Liver**	**Group 4 Female- Liver**
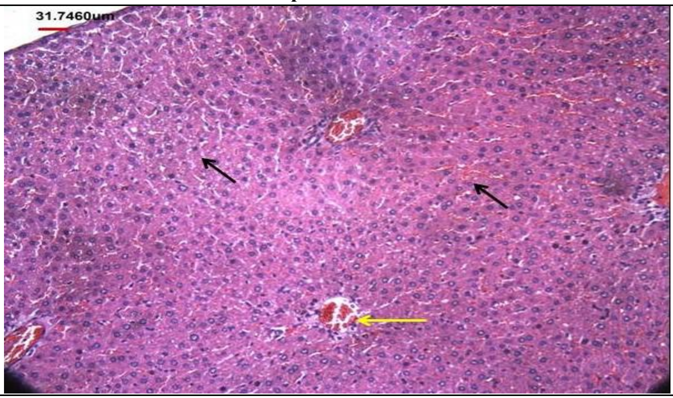	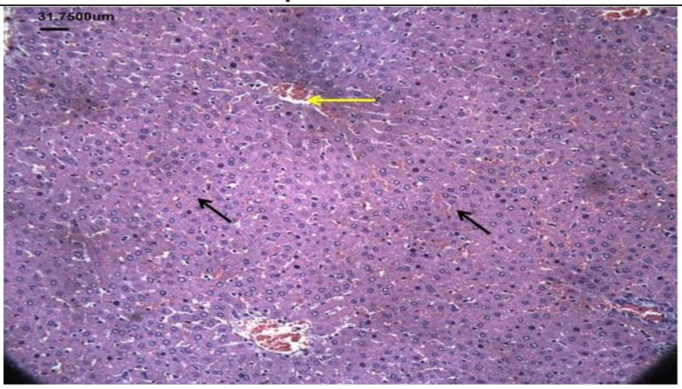
**Showing normal architecture of hepatocytes (arrow) with minimal congestion (yellow arrow).**	**Showing normal architecture of hepatocytes (arrow) with minimal congestion (yellow arrow).**

**Supplementary Figure 2:** Representative Histopathology images- H&E.X200 –Athymic Nude mice	
**Group 1 Male- Liver**	**Group 1 Female- Liver**
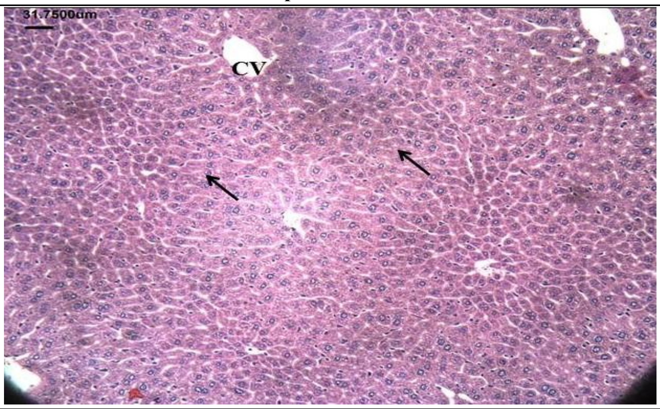	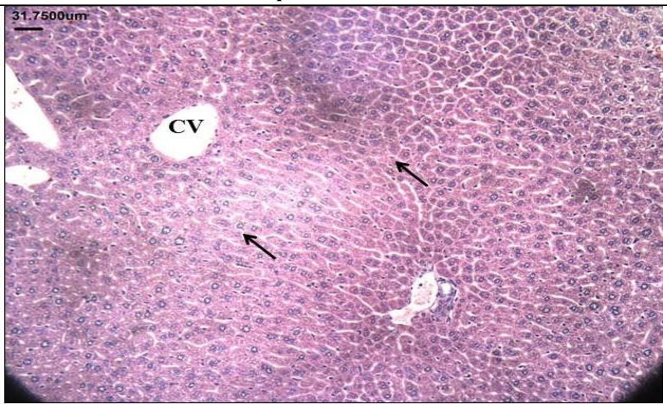
**Showing normal architecture of hepatocytes (arrow) and central vein (CV)**	**Showing Normal architecture of hepatocytes (arrow) and central vein (CV)**
**Group 4 Male- Liver**	**Group 4 Female- Liver**
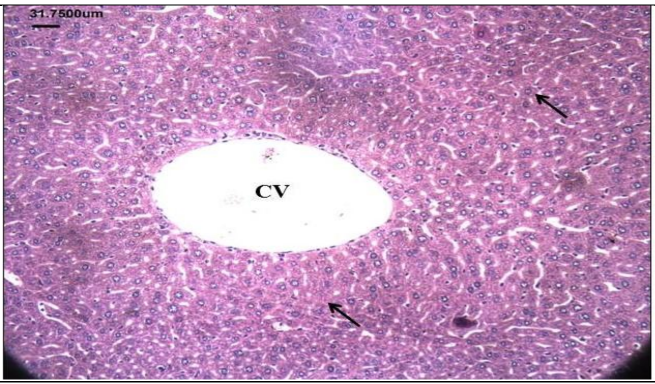	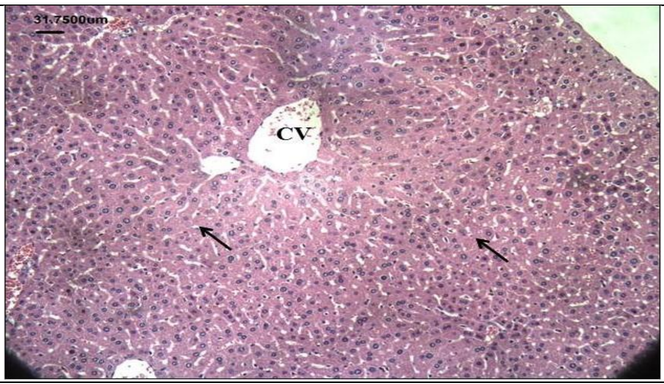
**Showing normal architecture of hepatocytes (arrow) and central vein (CV).**	**Showing normal architecture of hepatocytes (arrow) and central vein (CV).**
**Group 1 Male- Spleen**	**Group 1 Female-Spleen**
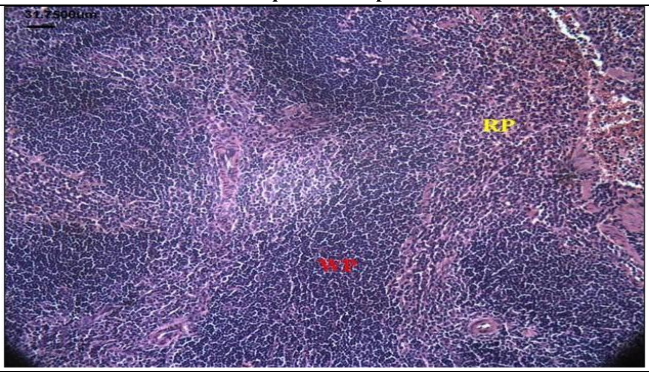	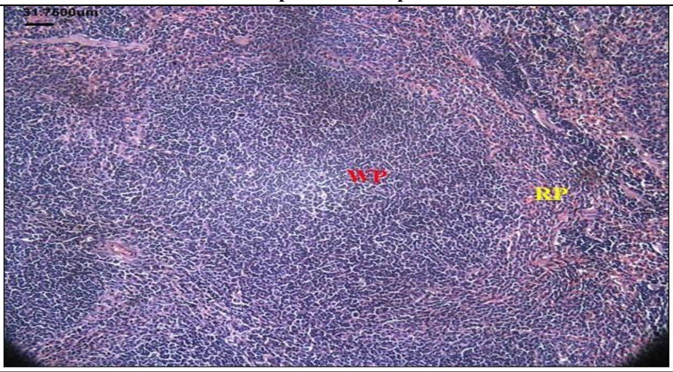
**Showing normal architecture of red pulp (RP) and white pulp (WP).**	**Showing normal architecture of red pulp (RP) and white pulp (WP).**
**Group 4 - *Male- Spleen***	**Group 4 Female- Spleen**
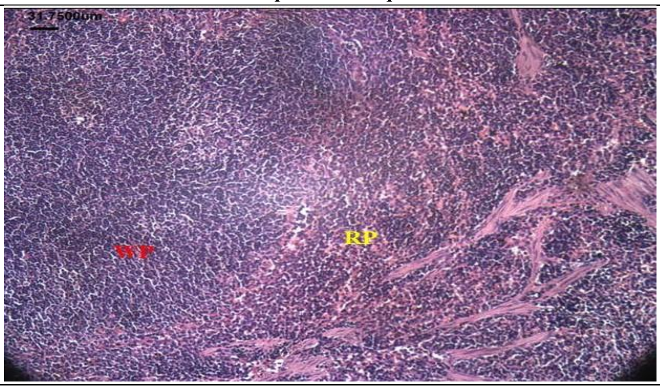	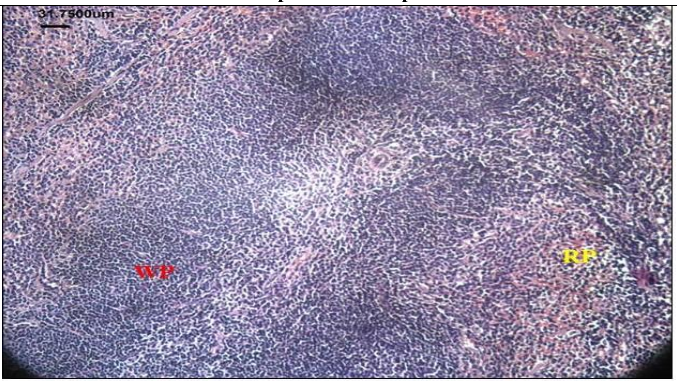
**Showing normal architecture of red pulp (RP) and white pulp (WP).**	**Showing normal architecture of red pulp (RP) and white pulp (WP)**
**Group 1 Male Intestine**	**Group 1 - Female Intestine**

**Supplementary Figure 2:** Representative Histopathology images- H&E.X200 –Athymic Nude mice	
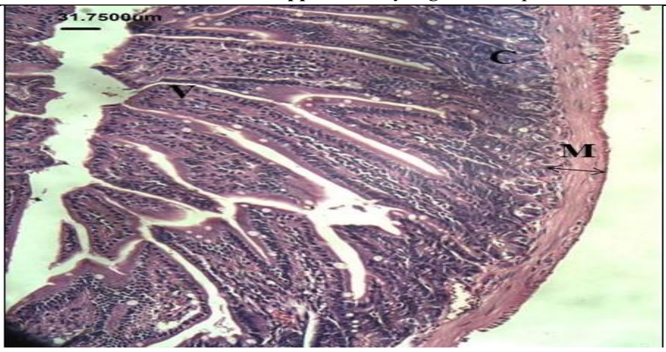	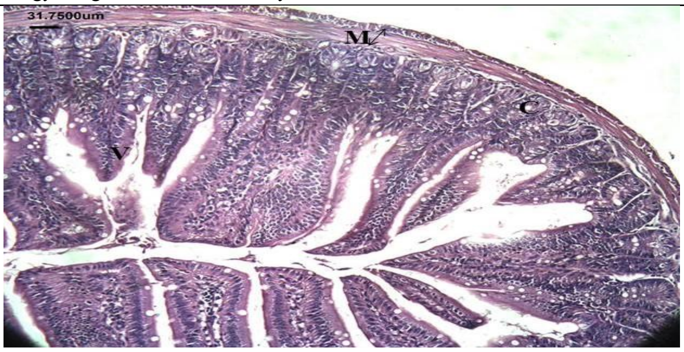
**Showing normal architecture of intestinal villi (V), crypt (C) and Muscularis (M).**	**Showing normal architecture of intestinal villi (V), crypt (C) and Muscularis (M).**
**Group 4 - Male Intestine**	**Group 4 Female Intestine**
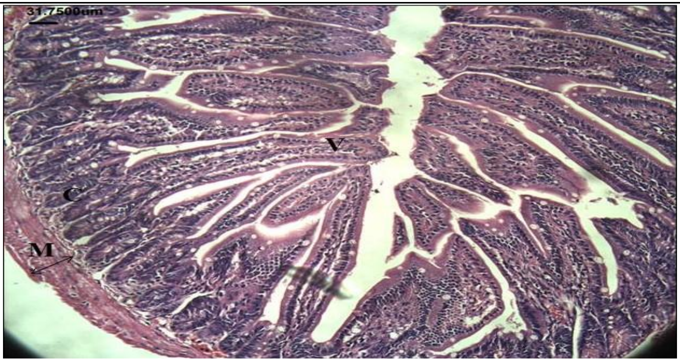	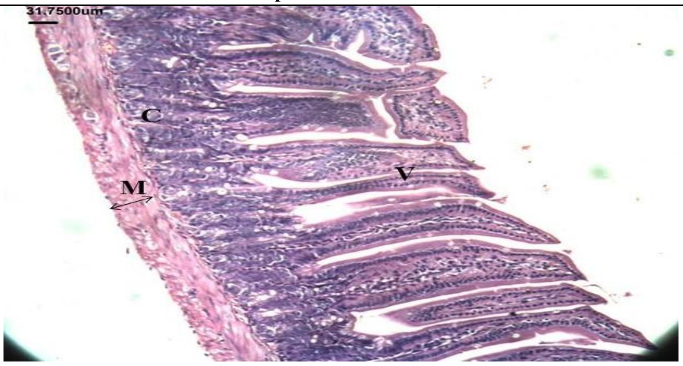
**Showing normal architecture of intestinal villi (V), crypt (C) and Muscularis (M).**	**Showing normal architecture of intestinal villi (V), crypt (C) and Muscularis (M).**
**Group 1 Male- Mesenteric lymph node**	**Group 1 Female - Mesenteric lymph node**
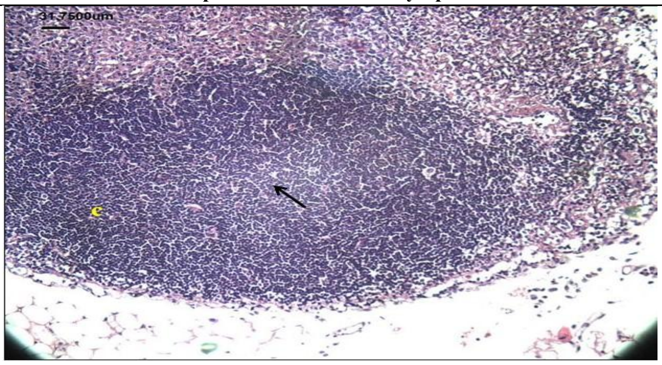	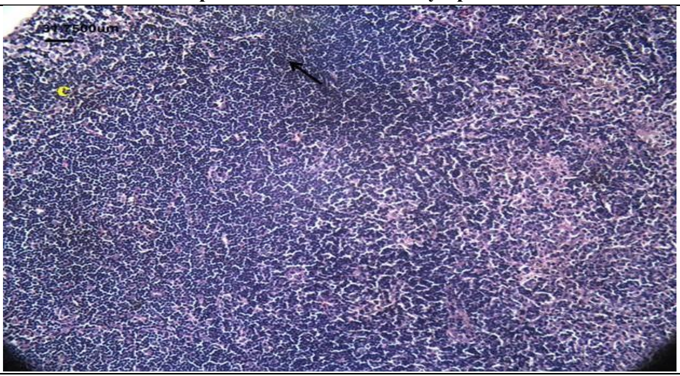
**Showing normal architecture of lymphoid follicle (arrow) and cortex (C).**	**Showing normal architecture of lymphoid follicle (arrow) and cortex (C).**
**Group 4 Male- Mesenteric lymph node**	**Group 4 Female - Mesenteric lymph node**
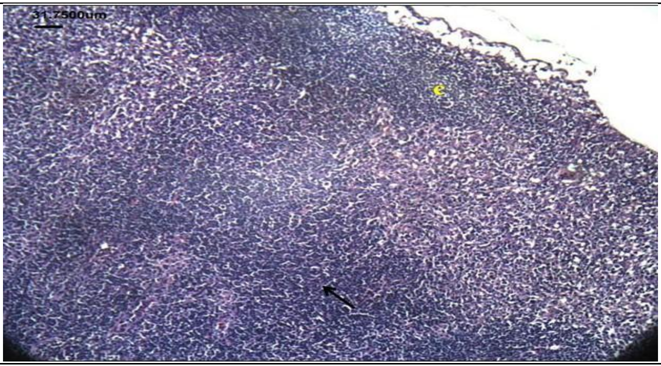	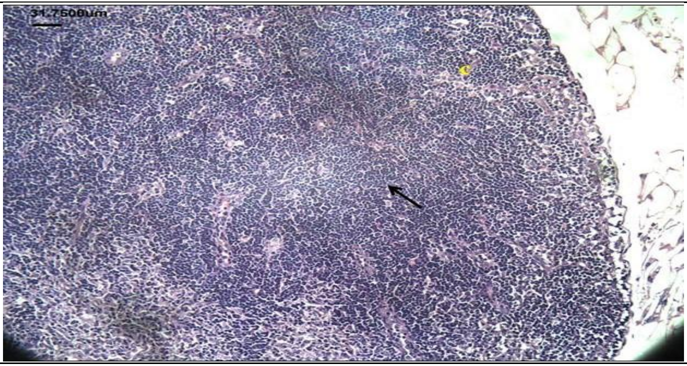
**Showing normal architecture of lymphoid follicle (arrow) and cortex (C).**	**Showing normal architecture of lymphoid follicle(arrow) and cortex (C).**
